# S100A8^+^S100A9^+^ transitional macrophages are associated with pulmonary fibrosis progression by integrating immunometabolism and fibrogenic crosstalk

**DOI:** 10.3389/fimmu.2026.1832940

**Published:** 2026-07-02

**Authors:** Censhan Lin, Mingyue Wu, Ziying Zhang, Muxu Zheng, Huiqin Li, Yufang Wang, Zhanxin Du, Linxiang Wu, Junjie Dai, Yu Yu, Xuhang Du, Yehui Wu, Jie Xu, Zhongxing Wang, Fu-Li Xiang

**Affiliations:** 1 Department of Anesthesiology, The First Affiliated Hospital, Sun Yat-sen University, Guangzhou, China; 2 Institute of Precision Medicine, the First Affiliated Hospital of Sun Yat-sen University, Guangzhou, China; 3 National Health Commission of the People's Republic of China (NHC) Key Laboratory of Assisted Circulation and Vascular Diseases, Sun Yat-sen University, Guangzhou, China; 4 Department of Radiation Oncology, The Affiliated Cancer Hospital of Xiangya School of Medicine, Central South University/Hunan Cancer Hospital, Changsha, Hunan, China; 5 State Key Laboratory of Oncology in South China, Cancer Center, Collaborative Innovation Center for Cancer Medicine, Sun Yat-sen University, Guangzhou, China; 6 Henan Academy of Innovations in Medical Science, Zhengzhou, Henan, China; 7 Department of Gynecology, The First Affiliated Hospital of Sun Yat-sen University, Guangzhou, China; 8 Department of Neurosurgery, The First Affiliated Hospital of Sun Yat-sen University, Guangzhou, China

**Keywords:** EGF/AREG signaling, glutamine metabolism/GLUL, idiopathic pulmonary fibrosis, macrophage heterogeneity, S100A8/S100A9, transitional macrophages

## Abstract

**Background:**

Idiopathic pulmonary fibrosis (IPF) is a progressive interstitial lung disease with limited therapeutic options. Although macrophage heterogeneity has been implicated in pathogenesis, the contribution of transitional macrophage states remains poorly understood.

**Methods:**

We integrated single−cell RNA−seq from 93 human lung specimens (44 IPF, 49 controls) and analyzed peripheral blood single−cell datasets (31 IPF, 17 controls). We examined differentiation trajectories and ligand–receptor networks and assessed metabolic programs. Functional relevance was evaluated in a bleomycin mouse model using histology, immunostaining, qRT−PCR, and tissue glutamine quantification.

**Findings:**

We identified an *S100A8^+^S100A9^+^* transitional macrophage population bridging *FABP4^+^MME^+^* precursors and *SPP1^+^MMP9^+^* effector macrophages. These cells are associated with fibrogenesis through two interconnected mechanisms: epidermal growth factor (EGF)-mediated signaling to fibroblasts and alveolar epithelial cells, amplifying profibrotic communication; and glutamine metabolic reprogramming, marked by *GLUL*. In murine bleomycin induced fibrosis, these features emerged early, with elevated *S100a8, S100a9, Fcna, Timp1*, and coincided with increased lung glutamine at day 14. Peripheral profiling confirmed concordant transcriptional changes in monocytes from IPF patients, supporting translational biomarker potential.

**Interpretation:**

*S100A8^+^S100A9^+^* macrophages might function as a druggable immune-metabolic hub in IPF. Targeting *S100A8/A9, GLUL*−dependent glutamine flux, or EGFR−axis signaling may enable actionable paths for mechanism−based therapy. Furthermore, their peripheral molecular signature may offer a disease-specific biomarker platform for early diagnosis and therapeutic monitoring.

## Introduction

Idiopathic pulmonary fibrosis (IPF), the most common form of pulmonary fibrosis, is characterized by progressive parenchymal scarring and a poor prognosis, with fewer than 30% of patients surviving beyond five years after diagnosis (1–4). Although its etiology remains incompletely understood, the pathogenesis of IPF is attributed to complex interactions between genetic susceptibility and microenvironmental triggers (5–8). Diagnosis currently relies primarily on high-resolution computed tomography (HRCT) to identify usual interstitial pneumonia (UIP) patterns, although surgical biopsy remains essential for atypical cases (7, 9–11). Therapeutic options remain limited: antifibrotic agents (pirfenidone and nintedanib) modestly slow functional decline but fail to reverse established fibrosis (2, 12–14). The refractory nature of the disease arises not only from fibrosis itself but also from dysregulated immune crosstalk.

Single-cell technologies have recently reshaped our understanding of macrophage roles in IPF, revealing their transition from inflammatory effectors to key regulators of fibrogenesis (15–17). Transcriptomic studies have identified distinct profibrotic macrophage subsets, including *PLA2G7^+^* populations that drive extracellular matrix deposition (18), *SPP1^+^* macrophages with proliferative dominance within fibrotic niches (19), and *TREM2^+^* subsets that activate pathogenic fibroblasts (20), but the transitional states and their metabolic–paracrine coupling remain incompletely resolved.

By integrating single-cell RNA sequencing data from 93 human samples (49 controls, 44 IPF) (21–27) and validating in a bleomycin model and peripheral blood, we identified a *S100A8^+^S100A9^+^* transitional macrophage subpopulation that might play a central role in IPF progression bridging *FABP4^+^MME^+^* and *SPP1^+^MMP9^+^* macrophages. These cells are enriched for AREG–EGFR signaling to fibroblasts/epithelium and show *GLUL*−linked glutamine metabolic reprogramming; markers are detectable in circulating monocytes from IPF patients (28, 29). Our findings elucidate transitional macrophages form a druggable immune−metabolic hub in IPF. Targeting *S100A8/A9*, *GLUL*–glutamine flux, or EGFR−axis signaling, and leveraging a peripheral monocyte signature may enable mechanism−based therapy and earlier disease monitoring.

## Methods

### Published data set acquisition and processing

We collected 49 normal control and 44 idiopathic pulmonary fibrosis (IPF) lung tissue samples from the GEO database (GSE136831 (21–23), GSE122960 (24), GSE159585 (25), GSE159354 (26, 27)) together with 17 normal and 31 IPF peripheral blood single-cell transcriptome datasets (GSE233844 (28), GSE264196 (29)). Anti-fibrotic treatment status was not uniformly available in the public metadata and associated source publications for all included datasets; therefore, we could not systematically determine or control for whether all samples were treatment-naïve. Single-cell RNA-seq data were analyzed with Seurat (v5.2.1) (18). Each dataset was first processed individually before integration using a common quality-control workflow. Cells were filtered out if they contained nCount_RNA <500, nCount_RNA > 30000, nFeature_RNA>6000, nFeature_RNA<300 detected genes,>20% mitochondrial transcripts. Doublets were removed with DoubletFinder (v2.0.6, https://github.com/chris-mcginnis-ucsf/DoubletFinder), resulting in 430,206 lung tissue cells and 211,246 peripheral blood cells retained for downstream analysis. Data normalization was carried out using LogNormalize, and batch effects were corrected with Harmony (v1.2.3) (30). We identified 3000 highly variable genes via FindVariableFeatures. Dimensionality reduction was conducted using PCA, t-SNE, and UMAP, followed by clustering with FindNeighbors and FindClusters. Cell identities were assigned based on canonical markers and expression patterns. Because the integrated lung datasets included both scRNA-seq and snRNA-seq data (GSE159585), we acknowledge that applying a unified quality-control framework may not fully account for modality-specific differences in transcript capture, particularly mitochondrial transcript abundance and nuclear versus cytoplasmic RNA composition. To evaluate the robustness of key macrophage populations and transcriptional programs, additional sensitivity analyses were performed with or without GSE159585. To ensure the reliability of our downstream analyses, we first evaluated the quality metrics of the macrophage populations across the integrated datasets. An assessment of the mitochondrial gene fraction revealed that macrophages from GSE159585 showed generally low mitochondrial gene percentages, with most cells remaining below 5%, supporting the high quality of the retained macrophage population. In contrast, GSE122960 and GSE136831 displayed slightly wider distributions but remained within acceptable quality thresholds (**Supplementary Figures 1**, **2A**).

1) Dimensionality reduction, clustering, and visualization

The top variable genes were used for PCA. The number of informative principal components was estimated using elbow plots and by inspecting gene loadings. Clustering was performed with FindNeighbors and FindClusters, and clusters were visualized in UMAP (RunUMAP, DimPlot).

2) Cell clustering and annotation

**Figure 1:**
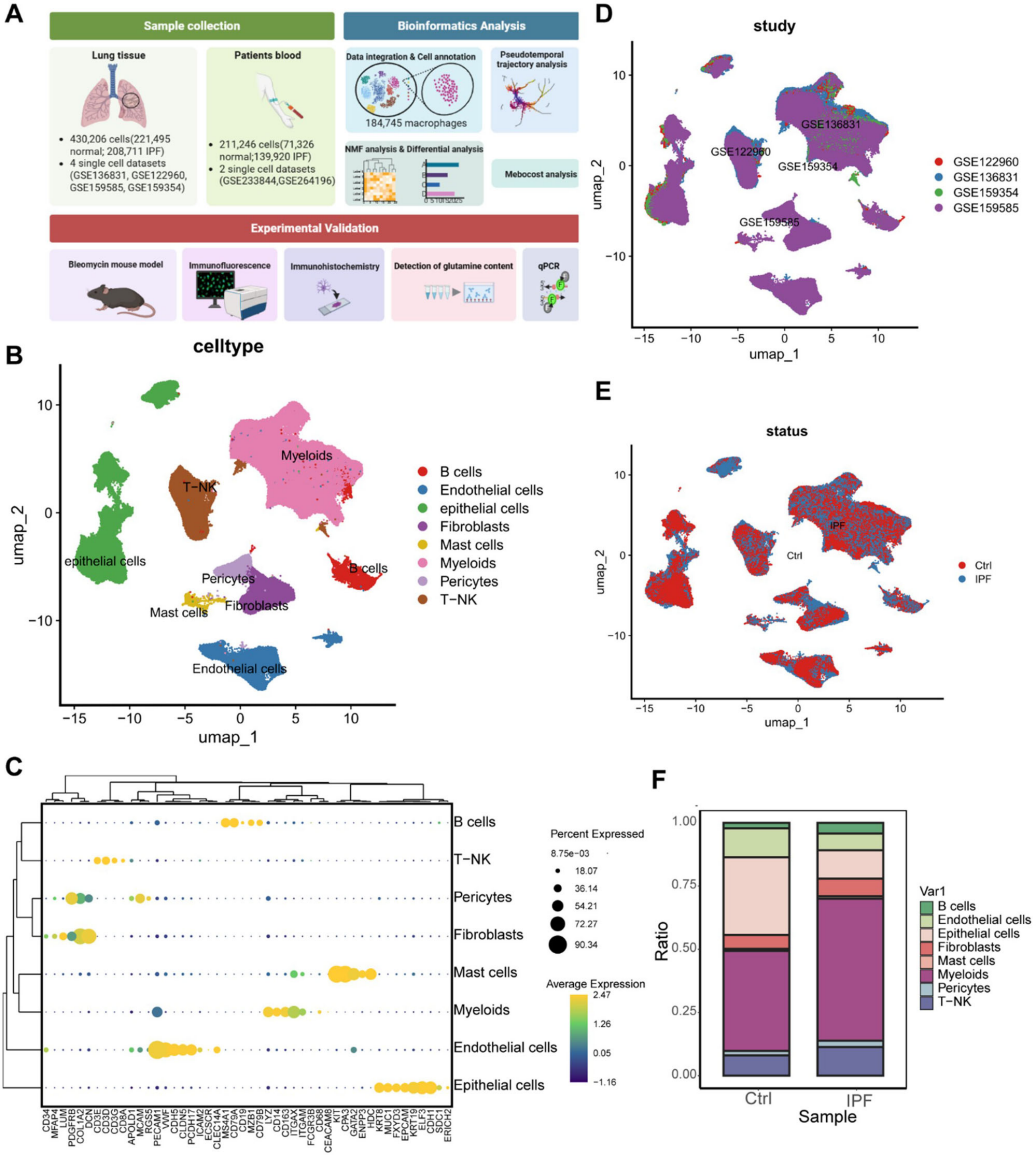
Single-cell transcriptomic atlas of Idiopathic Pulmonary Fibrosis (IPF). **(A)** Schematic overview of the study design, including sample collection from human lung and blood, the bioinformatics analysis pipeline, and the experimental validation workflow using a murine model. **(B)** UMAP projection of 430,206 cells passing quality control from 93 lung tissue samples, with transcriptionally distinct cell type clusters color-coded. **(C)** Dot plot showing the expression of representative marker genes used to annotate the identified cell clusters. Dot size corresponds to the percentage of cells expressing the gene, while color intensity represents the average expression level. **(D)** UMAP visualization of the integrated datasets, with cells colored by their study of origin, demonstrating robust alignment and removal of batch effects. **(E)** UMAP plots stratified by disease status, comparing the distribution of cells from control (Ctrl) and IPF samples. **(F)** Stacked bar chart showing the relative distribution of each cell cluster among captured cells in the control and IPF groups, illustrating differences in the composition of the sequenced cell populations between conditions.

Principal components with the highest variance were used for clustering with Seurat. UMAP projections were generated for visualization. Marker genes were identified with FindMarkers, and DEGs were extracted using FindAllMarkers, retaining only upregulated features. Cell types were annotated according to canonical markers (**Figure 1C**).

3) Identification of Macrophage Subpopulations

Macrophages, monocytes, and dendritic cells were subsetted from the integrated lung tissue single-cell dataset cell population described in Section 3.2 using the canonical markers. This yielded a total of 204,887 myeloid cells, among which 184,745 were identified as macrophages and used for subsequent subclustering. The subclustering analysis was performed following the same computational pipeline as described above (Section 3.2), utilizing 1,000 highly variable genes identified specifically within this macrophage subset. Cell identities of the resulting clusters were assigned based on established markers and distinct expression patterns.

4) Identification of macrophage subpopulations using GeneNMF

To assess macrophage heterogeneity, we applied GeneNMF (v0.7.0, https://github.com/carmonalab/GeneNMF). The top 1000 HVGs were selected after excluding cell-cycle genes. Macrophages were analyzed by non-negative matrix factorization (NMF) with sparsity constraints across multiple factorization ranks (k = 4–10). Each NMF factor was treated as an individual gene program, defined by genes with high weights. Recurrent gene programs identified across samples and NMF ranks were then compared based on their gene-weight profiles and aggregated into consensus metaprograms.

To systematically assess macrophage heterogeneity and extract reproducible biological signals, we applied GeneNMF analysis across the datasets (**Supplementary Figures 2B–D**; **Supplementary Table 1**). This approach successfully identified distinct macrophage metaprograms characterized by representative marker genes, including *FABP4^+^MME^+^, AREG^+^PID1^+^, SPP1^+^MMP9^+^*, and *S100A8^+^S100A9^+^*, among others. To confirm that these extracted metaprograms represent stable biological states rather than dataset-specific artifacts, we evaluated their robustness using mean similarity, silhouette score, and sample coverage metrics. The robustness analysis demonstrated that the key functional metaprograms, particularly MP3 (*FABP4*), MP5 (*S100A8/S100A9*), and MP6 (*SPP1*), maintained high similarity and silhouette scores both when assessed across three core datasets (GSE122960, GSE136831, GSE159354) and when expanded to include all four datasets (GSE122960, GSE136831, GSE159354, GSE159585).

To systematically define robust and recurrent gene expression programs within the macrophage compartment, we applied non-negative matrix factorization (GeneNMF) and evaluated consensus metaprograms across a range of factorization ranks (nMP = 4 to 10). The optimal number of metaprograms was determined by assessing three key robustness metrics: mean sample coverage, mean silhouette score, and mean similarity. As the number of consensus metaprograms increased, we observed a predictable, gradual decline in mean sample coverage, alongside a steady increase in mean similarity. Crucially, the mean silhouette score, which measures the cohesion and separation of the assigned gene programs, demonstrated an initial rise and reached a stable plateau at nMP=8 (**Supplementary Figure 3**). To achieve an optimal balance between maintaining broad sample representation and ensuring high program distinctness, nMP=8 was selected. This rank effectively resolved eight robust and transcriptionally discrete macrophage metaprograms (MP1-MP8) for subsequent biological annotation and downstream analysis.

5) Differential gene expression and peak analysis

Differential expression analysis was performed using the FindAllMarkers function for comparisons across all clusters and the FindMarkers function for direct pairwise comparisons between specified clusters. Genes were considered differentially expressed if they passed the following thresholds: an adjusted *p*-value < 0.05, a minimum log2-fold change of 1, and were detected in a minimum of 25% of cells in either of the two populations being compared (min.pct = 0.25). To enhance the stringency, we additionally required a minimum difference of 25% in the fraction of expressing cells between the two groups (min.diff.pct = 0.25) and pre-filtered features based on a log-fold change threshold of 0.25 (logfc threshold = 0.25).

6) Prediction of cellular plasticity using CytoTRACE

CytoTRACE (v1.1.0) (31) was used to calculate differentiation scores for individual cells based on gene expression diversity, with higher scores indicating greater stemness potential.

7) Monocle3 analysis

Pseudotime trajectories of macrophage subpopulations were reconstructed using Monocle3 (v1.3.7) (32). A principal graph was fitted to reduced-dimensional data, with root nodes defined by progenitor markers. Cells were ordered along pseudotime, and dynamic genes were identified using Moran’s I test (q < 0.05). Functional enrichment was performed via GO and KEGG. Trajectories were aligned with polarization markers (*NOS2, ARG1*) and validated using RNA velocity and *in vitro* assays.

8) Cell–cell communication analysis

Cell–cell interactions were explored using CellChat (v2.1.2) (33), focusing on ligand–receptor pairs where *S100A8^+^S100A9^+^* macrophages served as signaling sources.

9) Metabolite-mediated cell communication

Metabolite-driven communication was analyzed with MEBOCOST (v1.0.4) (34). Sender and receiver cells were defined by enzyme and sensor gene expression. Seurat objects were converted to Scanpy using SeuratDisk (v0.0.0.9021) and create_obj was used to generate MEBOCOST objects. Interactions were inferred with infer_commu under default parameters. To further constrain efflux and influx rates, COMPASS (35) which was run in a Python 3.8 environment, results (secretion.tsv, uptake.tsv) were integrated, and outputs were visualized with MEBOCOST plotting functions.

### Animal experiments

All animal experiments were approved by the Sun Yat-sen University Animal Care and Use Committee (SYSU-IACUC-2024-003105). Eight-week-old male C57BL/6 mice were used for *in vivo* experiments. Pulmonary fibrosis was induced by intratracheal bleomycin (BLM; 2 mg/kg, #HY-17565A-10mg, MCE) injection (20, 36, 37). Mice were sacrificed on days 14 and 28 for analysis. All mice were housed in a specific pathogen-free (SPF) facility on a 12-hour light/dark cycle with ad libitum access to food and water.

In accordance with SAGER (Sex and Gender Equity in Research) guidelines, we report that this study was conducted exclusively in male mice aged 8 weeks. This single-sex design was chosen to minimize biological variability associated with the estrous cycle in females and to avoid the known confounding immune modulation effects of female sex hormones in lung injury models, thereby reducing the total number of animals required to achieve statistical power. We acknowledge that this approach limits the direct generalizability of our findings.

### Sirius red staining

Sirius Red staining was performed using a Sirius Red Staining Kit (Servicebio, G1078) according to the manufacturer’s instructions. Paraffin-embedded tissue sections were deparaffinized in xylene and rehydrated through a graded ethanol series. The sections were immersed in Sirius Red Solution A and incubated in an oven at 65 °C for 30 min, followed by differentiation in Sirius Red solution B for 2 min and staining with Sirius Red solution C for 1 h at room temperature. After staining, the sections were dehydrated rapidly in absolute ethanol, cleared in xylene, and mounted with neutral resin mounting medium. Sirius Red-stained lung sections were imaged using an Olympus BX63F bright-field microscope (Olympus Corporation, Tokyo, Japan). For each specimen, 3–6 representative non-overlapping fields were randomly selected and captured at ×200 magnification under identical imaging settings. Collagen deposition was quantified using ImageJ software (Version 1.53, NIH, Bethesda, MD, USA). Sirius Red-positive areas were identified using a uniform threshold applied to all images. Large blood vessels, airway lumens, tissue folds, and other non-tissue regions were excluded from analysis before quantification. Collagen content was expressed as the percentage of Sirius Red-positive area relative to the total tissue area. The mean value from all analyzed fields was used for statistical analysis.

### Histological and molecular analysis

Paraffin-embedded lung sections were deparaffinized, underwent heat-induced antigen retrieval in a sodium citrate buffer, and incubated with 3% hydrogen peroxide to quench endogenous peroxidase. Sections were then blocked with a solution containing 5% donkey serum, 1% BSA, and 0.1% Triton-X100 before overnight incubation with primary antibodies at 4 °C followed by secondary antibody (ZSGB-BIO, PV-6000) and DAB detection. Nuclei were counterstained with hematoxylin. For immunofluorescence, deparaffinized sections underwent antigen retrieval and blocking before overnight incubation with primary antibodies at 4 °C followed by TSA (CY3, FITC, CY5, Alexa Fluor 594) conjugated secondary antibodies. Nuclei were counterstained with DAPI. Images were acquired with a confocal microscope (Olympus, FV3000). The primary antibodies used include anti-Fcna (Proteintech, 11775-1-AP, 1:100), anti-Timp1 (Proteintech, 16644-1-AP, 1:100), anti-S100a8 (Proteintech, 15792-1-AP, 1:100), anti-cd68 (CST, 97778S, 1:150), anti-CD68 (Bio-Rad, MCA1957, 1:200), anti-a-SMA (CST, 19245S, 1:320), anti-S100a9 (Proteintech, 26992-1-AP, 1:100), and anti-GLS (Proteintech, **6**6265-1-Ig, 1:100). Immunofluorescence images were acquired using an Olympus BX63F fluorescence microscope (Olympus Corporation, Tokyo, Japan) under identical exposure and acquisition settings for all experimental groups. For each specimen, 3–6 representative non-overlapping fields were randomly selected and imaged at the same magnification. Quantitative analysis was performed using QuPath software (Version 0.7.0, University of Edinburgh, Edinburgh, UK). Cells were automatically detected using the built-in cell detection algorithm based on nuclear staining, followed by fluorescence intensity-based classification. Positive cells were identified using predefined threshold settings that were applied uniformly across all samples. For co-localization analysis, the proportion of double-positive cells was calculated relative to the corresponding reference cell population. Threshold parameters were established before analysis and maintained throughout the study to minimize observer bias. For each specimen, measurements from all analyzed fields were averaged to obtain a single value for subsequent statistical analysis. All analysis was performed by an investigator blinded to the experimental groups.

### RNA extraction and qRT-PCR

Total RNA was extracted with TRIzol (Invitrogen, USA). cDNA was synthesized with the First-Strand cDNA Synthesis Kit (Invitrogen), and qPCR was performed with SYBR Green (Applied Biosystems) on an ABI 7500 system. Expression was normalized using the 2−ΔΔCt method, with technical triplicates.

### Glutamine quantification

Glutamine levels were measured with the Solarbio Glutamine Assay Kit (BC5300). Samples were deproteinized, centrifuged at 12,000 × g for 5 min at 4 °C, and supernatants were collected. Standards (0–100 μM) and samples were incubated with assay reagents at 37 °C for 1 h. Absorbance at 450 nm was measured, and concentrations were calculated from a standard curve. Cellular values were normalized to protein content.

### Statistical analysis

For animal experiments, quantitative data are presented as mean ± SEM. Statistical analyses were performed using GraphPad Prism software (v9.0, GraphPad Software). For comparisons between two groups with normally distributed data, an unpaired, two-tailed Student’s t-test was used. For comparisons between more than two groups with normally distributed data, a one-way ANOVA was performed, followed by Tukey’s multiple comparisons *post-hoc* test. A *p*-value < 0.05 was considered statistically significant. The specific tests used for each experiment are detailed in figure legends.

## Results

### Single-cell transcriptomic profiling defines major cellular compartments in IPF

To delineate the cellular architecture of idiopathic pulmonary fibrosis (IPF), we assembled and integrated single-cell RNA-seq data from four public lung tissue datasets (21–27), encompassing 49 healthy control and 44 IPF specimens (**Figure 1A**). The integration of the four lung tissue datasets created a cohesive cellular atlas, free of significant batch effects. To confirm the robustness of this integration at a granular level, we visualized the contribution of each of the 93 individual biological samples to the UMAP projection. This revealed a thorough intermixing of cells from different donors across all identified cell type clusters, confirming that the clustering was not driven by patient-specific effects (**Supplementary Figure 4A**). While the final integrated dataset was well-harmonized, we noted that the source datasets exhibited considerable heterogeneity in their original cellular composition. For instance, the GSE122960 dataset was predominantly composed of epithelial cells, whereas other datasets contained larger proportions of myeloid and T-cell populations (**Supplementary Figure 4B**). This underlying variability across studies highlights the importance of computational integration for generating a unified and comprehensive map of the IPF lung microenvironment.

After rigorous preprocessing and quality control (**Supplementary Figures 1**; **2**), a total of 430,206 high-quality lung tissue cells were retained for downstream analysis. Unsupervised graph-based clustering followed by UMAP dimensionality reduction resolved the principal cellular compartments of the lung, including epithelial cells, endothelial cells, fibroblasts, and various immune populations such as myeloid cells and T/NK cells (**Figure 1B**). The identity of these clusters was confirmed by the expression of canonical marker genes (**Figure 1C**). The datasets were robustly integrated, showing no significant batch effects, and cells from both control and IPF patients were distributed across all major lineages (**Figures 1D, E**).

A comparative assessment of the relative representation of transcriptionally defined cell populations among captured cells revealed marked alterations in IPF lungs. We observed a significant expansion of myeloid populations and a concurrent, notable depletion of the epithelial and endothelial compartments in IPF tissue relative to normal controls (**Figure 1F**). These findings underscore a profound reshaping of the pulmonary microenvironment in IPF, which is dominated by an imbalance between immune cell infiltration and the loss of structural cell populations. Given the prominent expansion of the myeloid compartment, we next focused on these cells to resolve the disease-associated heterogeneity in greater detail.

### Re-clustering of myeloid cells uncovers transcriptionally discrete macrophage subpopulations

**Figure 2:**
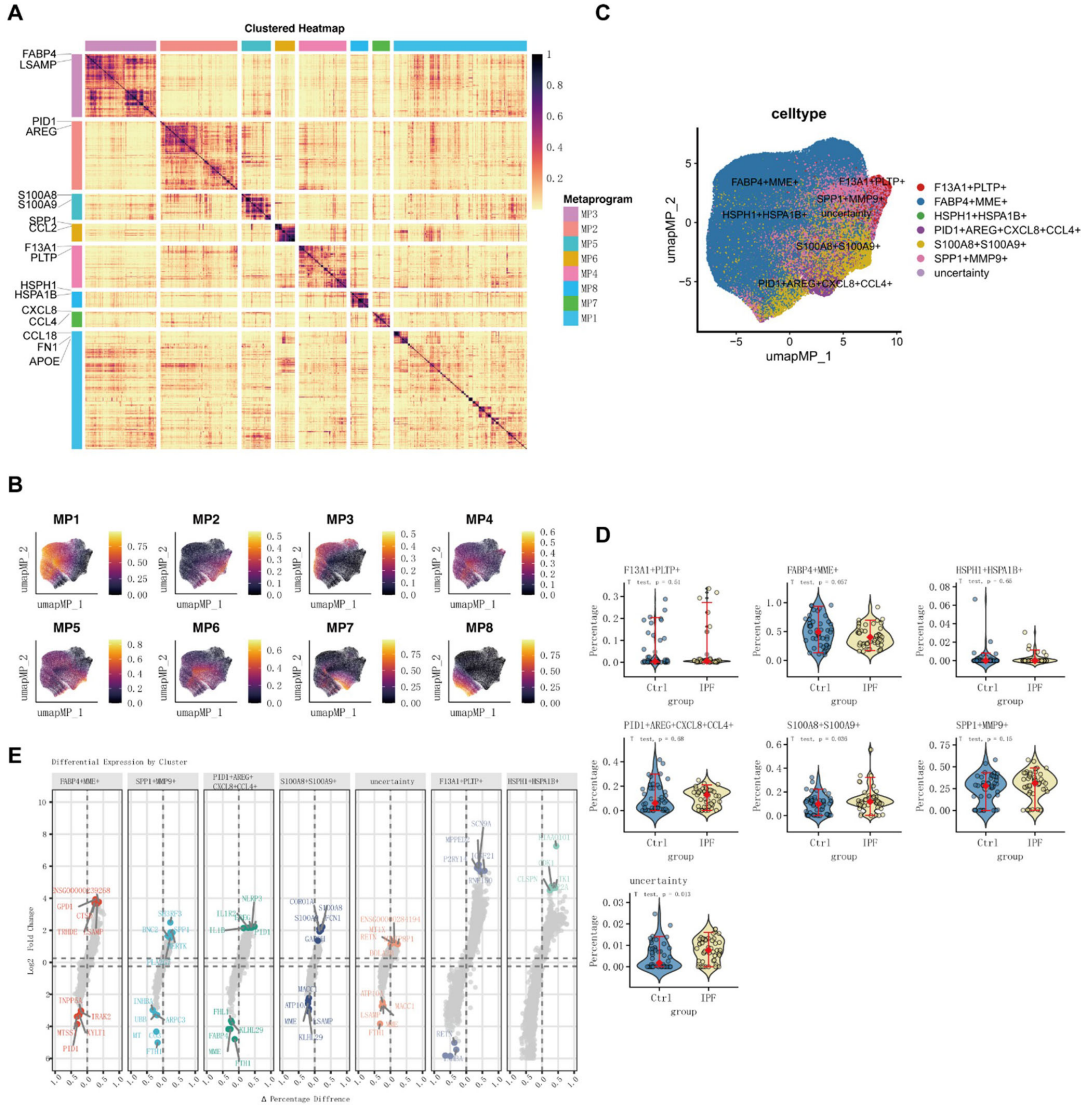
Intra-heterogeneity of macrophages in IPF. **(A)** Correlation heatmap of eight macrophage metaprograms identified by NMF analysis across four datasets. Hierarchical clustering groups the metaprograms based on their correlation, with representative genes for key modules listed. The color key indicates the correlation coefficient. **(B)** UMAP projections mapping the expression score and spatial distribution of each of the eight NMF metaprograms across the macrophage landscape. **(C)** UMAP projection showing the final classification of macrophage subpopulations based on NMF metaprogram expression profiles (top). The corresponding UMAP colored by the source dataset demonstrates robust data integration (bottom). **(D)** Violin and dot plots show the percentage of each macrophage subpopulation across all 93 samples. Each point represents one case, with blue indicating controls (n=49) and yellow indicating IPF (n=44). *p*-values from statistical comparisons are shown. **(E)** Volcano plots highlighting the most significantly variable genes enriched within each of the seven macrophage subpopulations, with key upregulated (red) and downregulated (blue/cyan) genes labeled.

To further dissect the heterogeneity within the expanded myeloid compartment, we first isolated these cells for dedicated sub-clustering. This initial step resolved the three major myeloid lineages: dendritic cells, monocytes, and macrophages (**Supplementary Figure 5A**). We then focused exclusively on the macrophage population for a more granular analysis. We applied non-negative matrix factorization (NMF) to 184,745 macrophages, which resolved eight robust and distinct gene expression programs, termed metaprograms (MP1–MP8). An initial high-resolution, graph-based clustering partitioned the macrophages into 14 distinct subgroups (**Supplementary Figure 5B**). To assign biological identities to these granular clusters, we scored each subgroup based on the eight NMF-derived metaprograms. This revealed that the metaprograms were differentially active across the 14 subgroups (**Supplementary Figure 5C**), allowing us to consolidate them into seven functionally coherent and transcriptionally discrete subpopulations. A correlation heatmap demonstrated that these metaprograms represented discrete, co-regulated gene modules, with key marker genes including *FABP4* for MP3, *S100A8* and *S100A9* for MP5, and *SPP1* for MP6 (**Figure 2A**). Each metaprogram showed a distinct spatial distribution across the UMAP projection of all macrophages, confirming their localization to specific cell clusters (**Figure 2B**).

Based on the dominant metaprogram scores, we partitioned the macrophages into seven transcriptionally discrete subpopulations, including *FABP4^+^MME^+^*, *S100A8^+^S100A9^+^*, and *SPP1^+^MMP9^+^* macrophages as well as an uncertainty subpopulation with no shared transcriptional feature or dominant enrichment for any of the identified metaprograms (**Figure 2C**). Accordingly, this population could not be confidently assigned to a specific metaprogram-defined identity under our current analytical framework. This detailed workflow culminated in the final annotated map of macrophage subpopulations used for all downstream analyses (**Supplementary Figure 5D**). A quantitative assessment of cell proportions revealed a disease-associated remodeling of the macrophage landscape. Compared to controls, IPF lungs showed a marked expansion of *S100A8^+^S100A9^+^* macrophages and the uncertainty subpopulation (**Figure 2D**). Finally, differential expression analysis for each subset identified the top up- and down-regulated genes that provide unique molecular signatures for these distinct macrophage states (**Figure 2E**). Together, these data highlight a profound remodeling of the macrophage compartment in IPF, characterized by the enrichment of a pro-inflammatory population. To determine how these macrophage states are related to one another, we next examined their potential developmental trajectories.

### Trajectory analysis defines a transitional macrophage state in IPF

**Figure 3:**
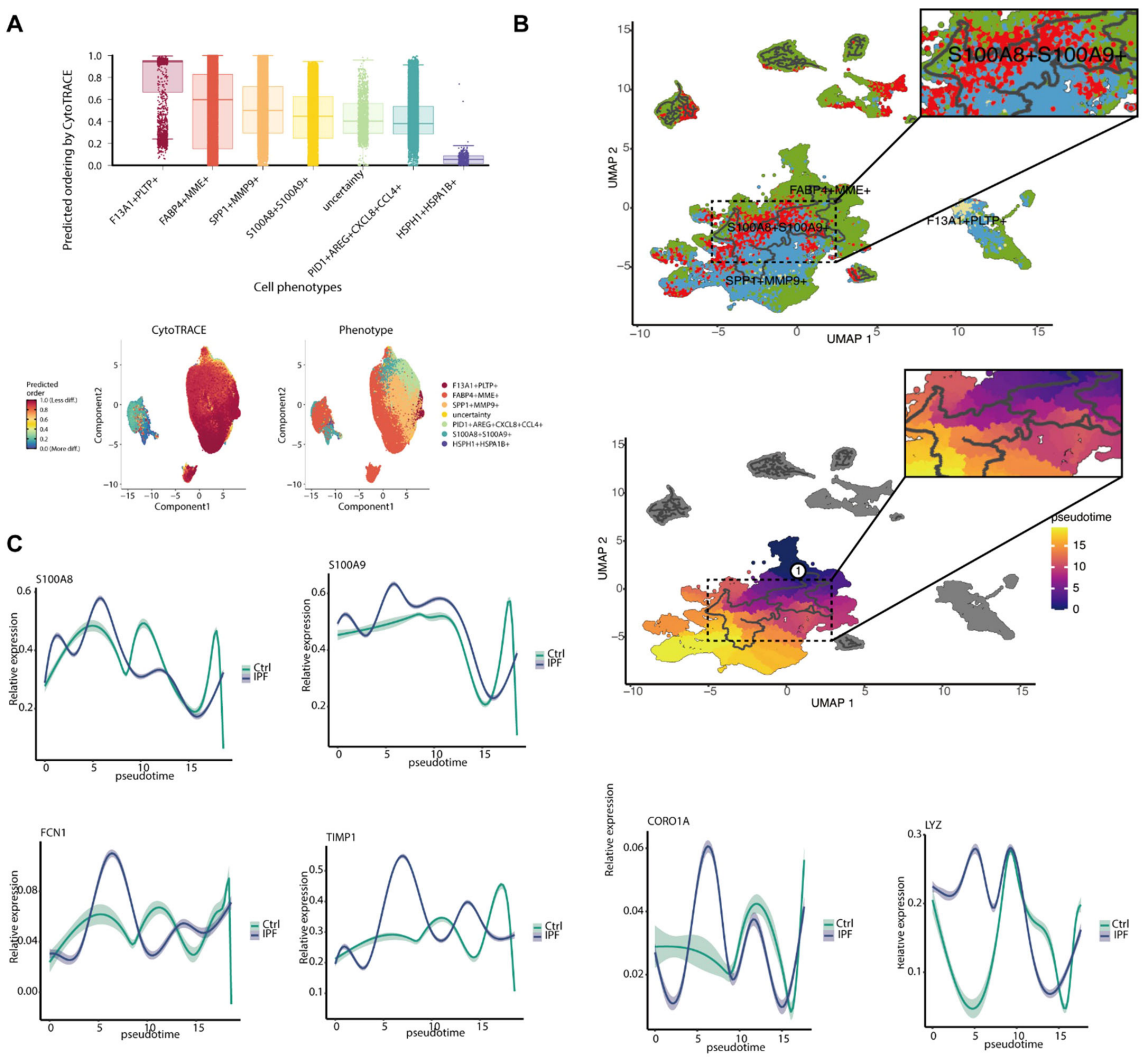
S100A8^+^S100A9^+^ macrophages act as a transitional hub driving fibrotic progression in IPF. **(A)** Stemness scores of eight macrophage subpopulations as assessed by CytoTRACE. The box plot (top) shows the distribution of predicted differentiation potential scores, while the UMAPs (bottom) project these scores onto the cellular landscape. **(B)** Monocle3 trajectory inference of macrophage differentiation. The top UMAP shows the principal graph connecting the subpopulations. The bottom UMAP is colored by pseudotime, illustrating a developmental progression originating from *FABP4^+^MME^+^* macrophages (dark purple). **(C)** Two-dimensional plots showing the relative expression scores for key transitional markers (*S100A8, S100A9, FCN1, TIMP1, CORO1A, and LYZ*) in control (green) and IPF (blue) samples along the pseudotime axis.

To investigate the developmental plasticity and relationships between the identified macrophage subpopulations, we first assessed their differentiation potential using CytoTRACE. This analysis revealed that the *F13A1^+^PLTP^+^*, *FABP4^+^MME^+^*, *S100A8^+^S100A9^+^*, and *SPP1^+^MMP9^+^* clusters exhibited high stemness scores, suggesting they exist along a dynamic differentiation continuum (**Figure 3A**). To reconstruct this developmental pathway, we applied Monocle3 pseudo-temporal trajectory analysis. The inference revealed a continuous trajectory originating from *FABP4^+^MME^+^* macrophages, which progressed through a distinct *S100A8^+^S100A9^+^* intermediate state before culminating in terminally differentiated *SPP1^+^MMP9^+^* macrophages (**Figure 3B**). This computationally derived trajectory is consistent with a model of clinical progression where precursor-like macrophages differentiate into pro-fibrotic effectors (38, 39).

Inspection of gene expression dynamics along the pseudotime axis further defined the molecular signature of this transition. Key genes expressed by *S100A8^+^S100A9^+^* macrophages, including *S100A8, S100A9, FCN1, TIMP1, CORO1A* and *LYZ* were markedly and transiently upregulated, with their expression peaking specifically at the intermediate *S100A8^+^S100A9^+^* state. We also traced mechano-sensing protein expressions including *PIEZO1/2, GPR68, NINJ1* and *TMEM63A/B/C* as mechanical environmental features have been shown to regulate immune response sensitivity and fibrosis progression. Interestingly, *NINJ1, PIEZO2* and *TMEM63A/B/C* were transiently upregulated at early stage (**Supplementary Figure 6**), overlapping the *S100A8^+^S100A9^+^* state. Importantly, this dynamic upregulation was a feature of the IPF trajectory and was significantly attenuated in control samples (**Figure 3C**). These data collectively establish that *S100A8^+^S100A9^+^* macrophages represent a pivotal transitional hub that associates with macrophage plasticity and fibrotic progression in IPF. Having identified this transitional macrophage program in lung tissue, we next asked whether related signatures could also be detected in the peripheral circulation.

### Peripheral blood profiling links monocyte expansion to transitional macrophages in IPF

**Figure 4:**
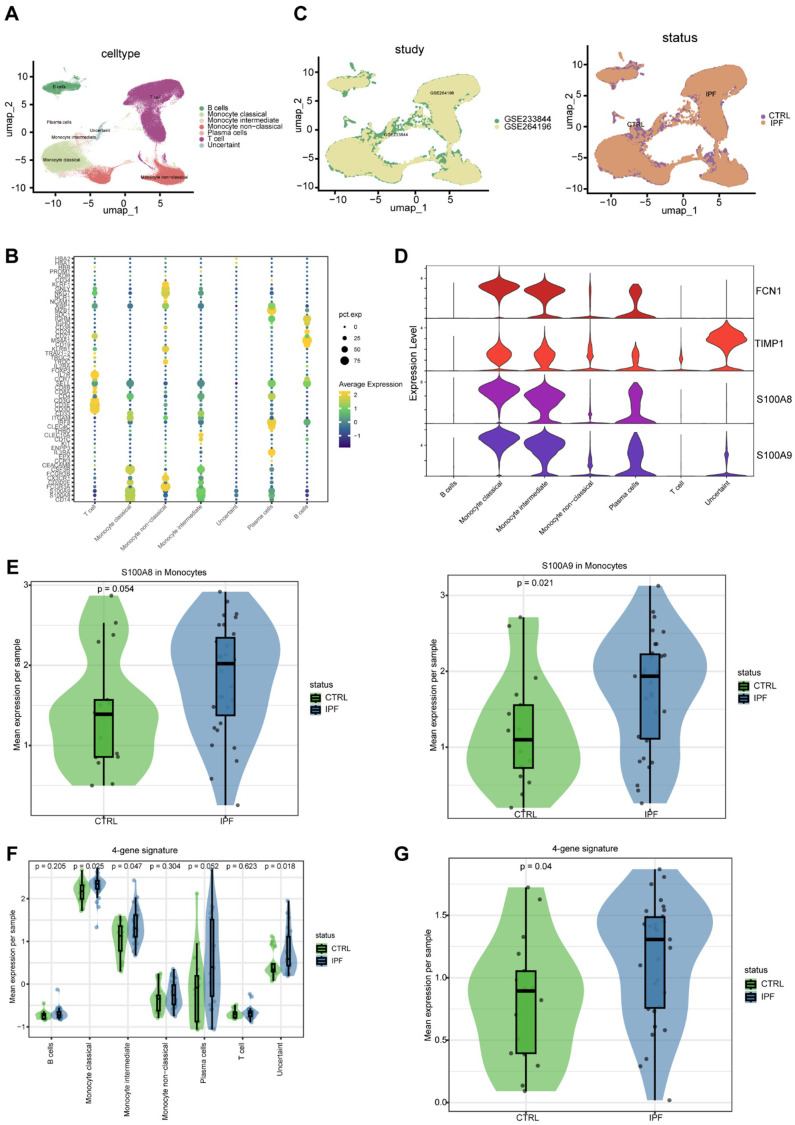
Peripheral blood monocyte biomarkers (*S100A8, S100A9, FCN1, TIMP1*) mirror lung macrophage dynamics. **(A)** UMAP projection of peripheral blood cells with distinct cell types labeled by color. **(B)** Dot plot showing canonical marker gene expression used to annotate the identified cell clusters. **(C)** UMAP visualization of peripheral blood cells stratified by dataset (left) or disease state (control vs. IPF, right), demonstrating successful data integration. **(D)** Violin plots display the expression levels of the four key transitional markers across the major cell populations. **(E)** Violin plots with boxplots show the mean per-sample expression of S100A8 and S100A9 in peripheral blood monocytes from Ctrl (n = 17) and IPF (n = 31) subjects. Each dot represents one sample. **(F)** Violin plots with boxplots show the mean per-sample expression of the 4-gene signature (*S100A8, S100A9, FCN1*, and *TIMP1*) across peripheral blood cell populations in Ctrl and IPF subjects. Each dot represents one sample. **(G)**Violin plots with boxplots show the mean per-sample expression of the 4-gene signature (*S100A8, S100A9, FCN1*, and *TIMP1*) in peripheral blood monocytes from Ctrl and IPFsubjects. Each dot represents one sample. Statistical testing was performed using the sample as the unit of inference, and the analysis is presented as exploration.

To determine whether the alterations observed in lung macrophages are detectable in the systemic circulation, we profiled peripheral blood single-cell transcriptomes from 31 IPF patients and 17 healthy donors (28, 29). Unsupervised clustering of the integrated dataset resolved the major peripheral blood mononuclear cell (PBMC) populations, including B cells, T cells, and distinct monocyte subsets (classical, intermediate, and non-classical), as well as an “uncertain” cluster that showed low canonical lineage-marker expression and therefore could not be confidently annotated despite modest S100A8/S100A9 expression (**Figure 4A**. **Figure 4B**). The data from all individual donors and studies were well-integrated, ensuring the robustness of our findings (**Figure 4C**; **Supplementary Figure 7A**).

A comparative analysis of cellular composition revealed a marked expansion of classical monocytes in the peripheral blood of IPF patients relative to healthy controls (**Supplementary Figure 7B**). We next evaluated the expression of the candidate markers identified from the lung tissue analysis. The transitional macrophage-associated genes—*S100A8, S100A9, FCN1, TIMP1*—were predominantly enriched in classical and intermediate monocytes within the PBMC compartment (**Figure 4D**). When reanalyzed at the subject/sample level to avoid pseudoreplication, monocytes from IPF patients still showed significantly increased expression of *S100A8* and *S100A9* compared with those from healthy controls, and a composite 4-gene monocyte signature (*S100A8, S100A9, FCN1*, and *TIMP1*) showed a concordant increase in IPF (**Figure 4E**). The individual results for *FCN1* and *TIMP1*, as well as the corresponding analyses in other peripheral blood cell populations, are provided in **Supplementary Figures 8**, **9**. These peripheral blood findings are presented as exploratory supportive evidence that the lung-associated monocyte/macrophage program may also be detectable in circulation, rather than as independently validated translational biomarkers. To further assess the biological relevance of this transitional program beyond transcriptomic inference, we next examined its temporal induction in an *in vivo* model of pulmonary fibrosis.

### *In vivo* validation confirms early induction of transitional macrophage markers in pulmonary fibrosis

**Figure 5:**
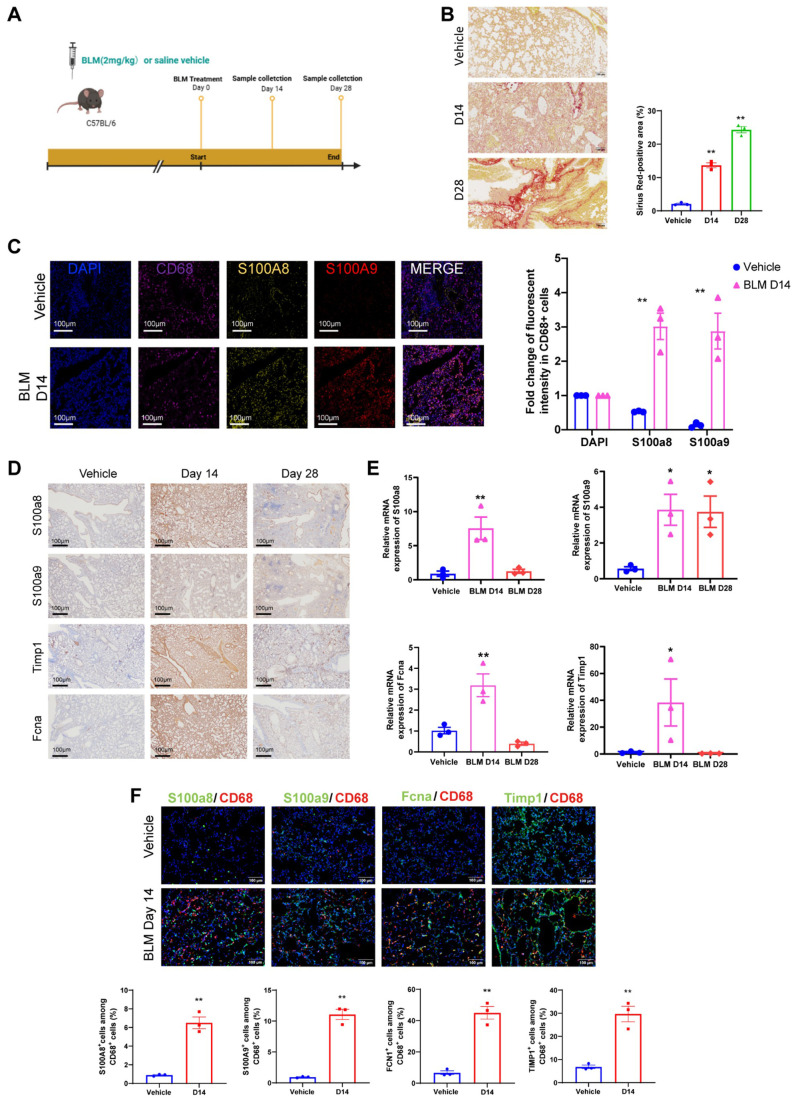
Convergent upregulation of fibrosis-associated mediators (S100a8, S100a9, Fcna, Timp1) in early-stage bleomycin-induced pulmonary fibrosis. **(A)** Schematic of the experimental design for the murine bleomycin (BLM) lung injury model, with tissue collection at day 14 and 28. **(B)** Representative images and quantification of lung tissue fibrosis treated with Vehicle, and BLM day 14 and day 28 post-bleomycin injury. Staining shows Sirus Red representing tissue fibrosis. Scale bars: 100 μm. **(C)** Representative immunofluorescence images of lung tissues treated with Vehicle and day 14 post-bleomycin injury. Staining shows *S100a8* (yellow), *S100a9* (red), and the macrophage marker Cd68 (magenta). Nuclei are counterstained with DAPI (blue). Scale bars: 100 μm. Fluorescent signal was quantified and compared between groups. **(D)** Longitudinal immunohistochemical analysis of *S100a8, S100a9, Fcna* and *Timp1* protein expression in lung tissues across the 28-day time course. Brown staining indicates positive signal. Scale bars: 100 μm. **(E)** Quantitative RT-PCR analysis of *S100a8, S100a9, Fcna* and *Timp1* mRNA levels in lung tissues at the indicated time points. **(F)** Representative immunofluorescence images of lung tissues at day 0 and day 14 post-bleomycin injury. Staining shows *S100a8* (greenw), *S100a9* (green), *Fcna* (green) and *Timp1*(green), and the macrophage marker Cd68 (red). Nuclei are counterstained with DAPI (blue). Fluorescent signal was quantified and compared between groups. Scale bars: 100 μm. N = 3 mice per group. Data are presented as mean ± SEM. *p < 0.05, **p < 0.01 versus Vehicle.

To validate our single-cell findings *in vivo* and characterize the temporal expression of the transitional macrophage signature, we established a bleomycin-induced pulmonary fibrosis model in mice (**Figure 5A**). The lung fibrosis induced by bleomycin was confirmed by Sirus red staining (**Figure 5B**). Immunofluorescence staining of lung tissue revealed a robust upregulation of *S100a8* and *S100a9* proteins on day 14 post-injury (**Figure 5C**). Importantly, this signal showed strong co-localization with the macrophage marker Cd68, confirming that macrophages are a primary source of these proteins during the early inflammatory response.

We further assessed the protein expression of all four key markers—*S100a8, S100a9*, *Fcna*, and *Timp1* on day 14 and 28 after injury using immunohistochemistry. This analysis demonstrated a consistent and marked elevation of all markers, with expression peaking at day 14, which corresponds to the critical inflammatory and early fibrotic phase of the model (**Figure 5D**). Quantitative RT-PCR of whole lung homogenates showed a significant increase in mRNA levels for *S100a8, S100a9, Fcna*, and *Timp1*, again peaking at day 14 (**Figure 5E**). Co-staining of CD68 with these four markers confirmed their specific increased expression in macrophages (**Figure 5F**). Together, these *in vivo* data validated our discoveries from human tissue, supporting the role of the transitional macrophage program as an early marker of fibrotic remodeling. We then sought to identify the potential signaling pathways through which this macrophage state may influence the fibrotic microenvironment.

### Transitional macrophages are predicted to drive paracrine signaling via the AREG–EGFR axis

**Figure 6:**
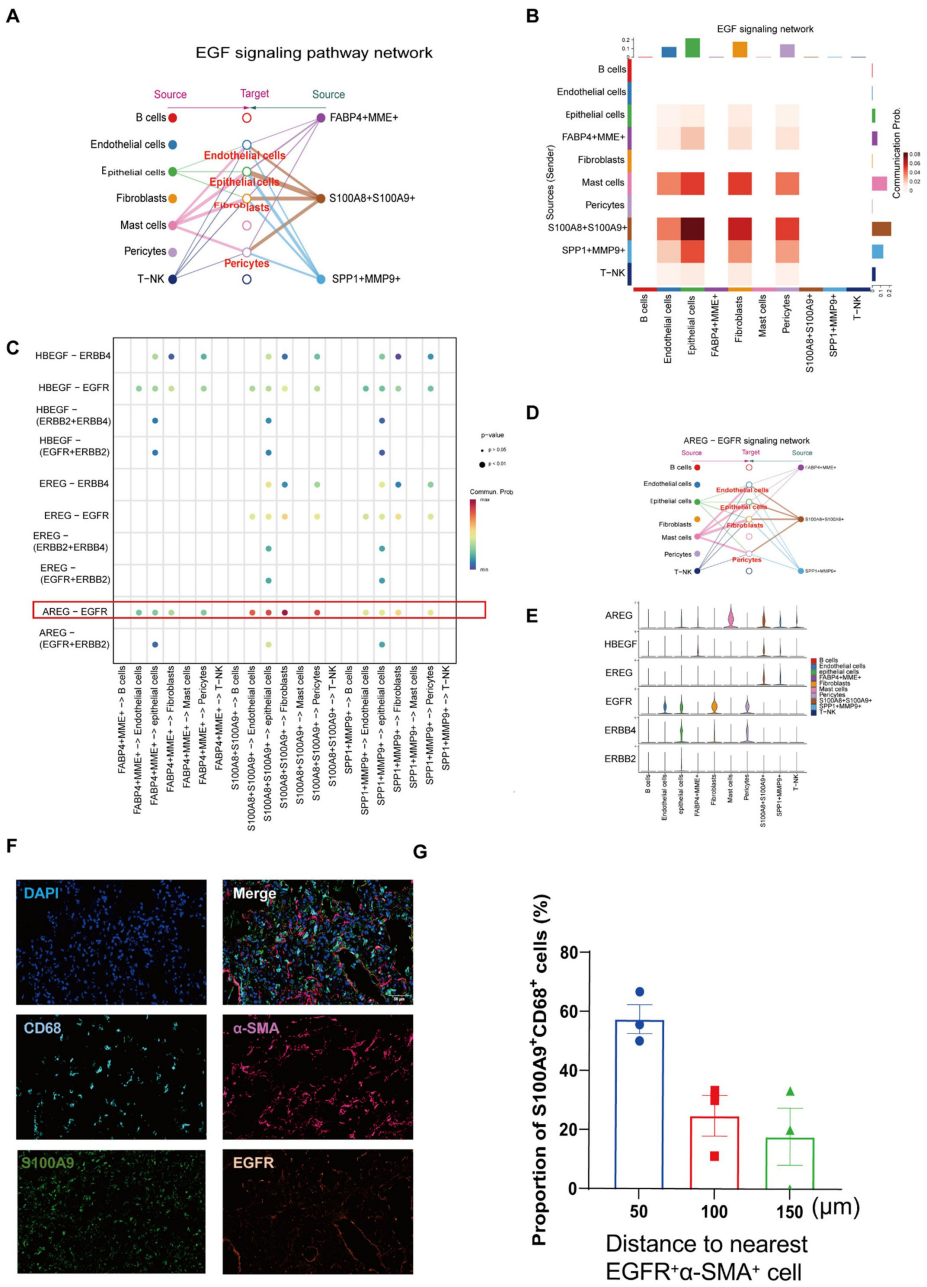
S100A8^+^S100A9^+^ macrophages drive IPF progression via AREG–EGFR paracrine signaling. **(A)** CellChat circular plot showing the overall EGF signaling network, with arrows indicating the direction and strength of communication from sender to receiver cells. **(B)** Heatmap inferred the interaction strength of the EGF signaling network between different cell populations. **(C)** Network plot predicted significant ligand–receptor pairs within the EGF signaling pathway. **(D)** Circular plot inferred the specific communication mediated by the AREG–EGFR pair, with S100A8^+^S100A9^+^ macrophages as the dominant source. **(E)** Violin plots showing the expression of key EGF pathway ligands (AREG, HBEGF, EREG, EGF) and the receptor (EGFR) across macrophage subpopulations. **(F)** Representative multiplex immunofluorescence image of mouse lung tissue day 14 post-bleomycin treatment, showing S100a9 (green), the macrophage marker Cd68 (cyan), the fibroblast marker α-SMA (magenta), and Egfr (red). Scale bar: 50 μm **(G)** Representative multiplex immunofluorescence image of mouse lung tissue at day 14 post-bleomycin treatment used for spatial proximity analysis, showing S100a9 (green), the macrophage marker Cd68 (cyan), the fibroblast marker α-SMA (magenta), and Egfr (red). Scale bar: 100 μm. Quantification of the proportion of S100A9^+^CD68^+^ cells in proximity to EGFR^+^αSMA^+^ cells confirmed the spatial proximity between these two cell populations.

To uncover the mechanisms by which *S100A8^+^S100A9^+^* transitional macrophages may promote fibrosis, we investigated their intercellular communication networks using CellChat. A global analysis identified macrophages and fibroblasts as prominent signaling hubs in the lung microenvironment, with a higher overall inferred in overall communication strength in IPF lungs compared to controls (**Figure 6A**). Pathway-level analysis revealed that the epidermal growth factor (EGF) signaling pathway was enriched in *S100A8^+^S100A9^+^* macrophages relative to several other cell populations (**Supplementary Figures 10A, B**). *S100A8^+^S100A9^+^* macrophages emerged as the primary source of these signals, with predicted communication directed toward fibroblasts, epithelial cells, endothelial cells and pericytes (**Figure 6B**).

Further dissection of this pathway identified the Amphiregulin (AREG) to EGFR ligand-receptor pair as the dominant interaction (**Figure 6C**). Transcriptomic analysis confirmed that AREG was highly and specifically expressed by the S100A8^+^S100A9^+^ macrophage subpopulation, while its receptor, EGFR, was broadly expressed across target structural cells, including fibroblasts and epithelial cells (**Figures 6D, E**). We performed multiplex immunofluorescence on lung tissue from our bleomycin mouse model. This analysis showed a spatial proximity between *S100a8^+^/S100a9^+^* macrophages and *α-SMA^+^/Egfr^+^* myofibroblasts at day 14 post-injury (**Figure 6F**). Although these imaging data do not directly validate ligand-receptor interactions or cell-type specificity, they are consistent with a potential contribution of predicted AREG-EGFR signaling within the macrophage-fibroblast niche to fibrotic progression. In parallel with intercellular signaling, we next examined whether this transitional macrophage state also displayed distinctive metabolic features.

### S100A8^+^S100A9^+^ transitional macrophages undergo glutamine metabolic reprogramming

**Figure 7:**
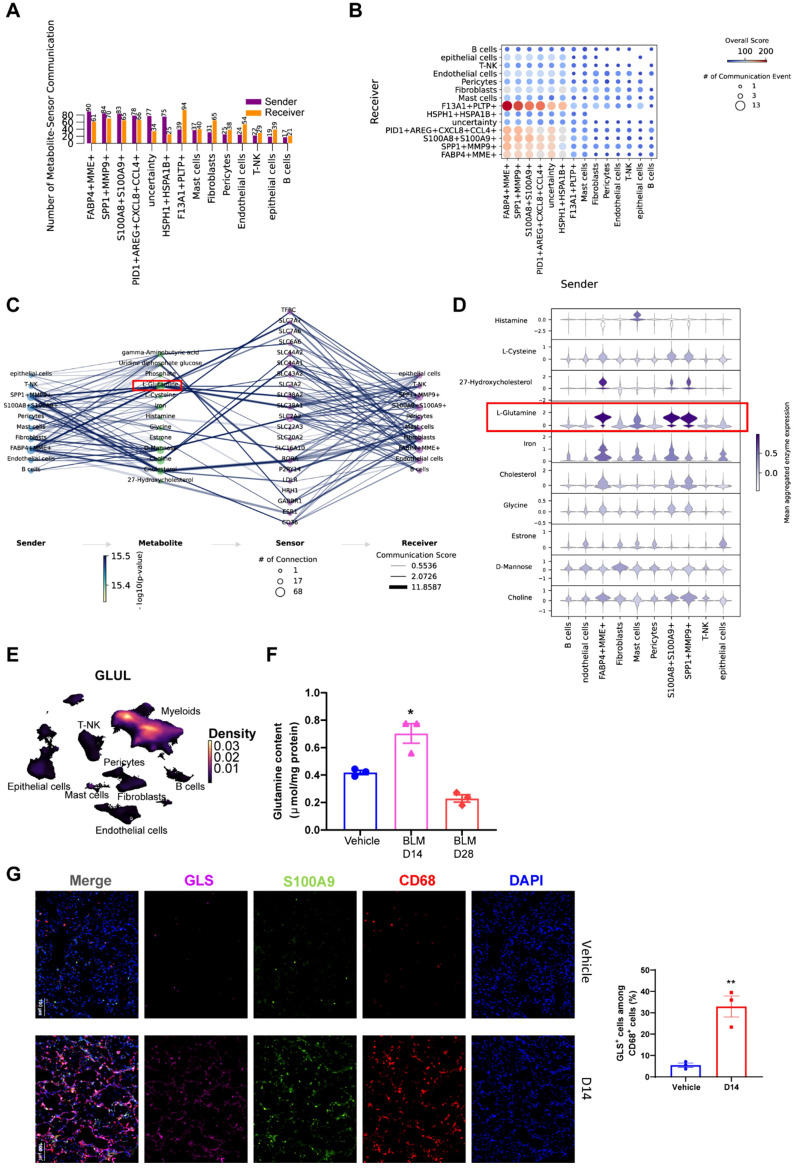
S100A8^+^S100A9^+^ transitional macrophages exhibit metabolic reprogramming and metabolite-mediated communication. **(A)** Quantitative analysis of metabolite-mediated communication: Bar chart showing the total number of incoming (receiver) and outgoing (sender) metabolite-sensor interactions across lung cell populations. **(B)** Global metabolite signaling heatmap: Dot plot visualizing the overall communication scores between sender and receiver cell types. The size of the dots represents the number of communication events, and the color represents the overall score. **(C)** Metabolite-sensor interaction network: Chord-style diagram illustrating specific connections between sender cells, secreted metabolites, and receiver sensors. L-Glutamine (red box) is identified as a key metabolite bridging various cell types. **(D)** Cell-specific metabolic enzyme expression: Violin plots showing the mean aggregated enzyme expression for various metabolic pathways. L-Glutamine metabolism (red box) is also detectable in *FABP4^+^MME^+^* precursor macrophages and appears more prominently enriched in *S100A8^+^S100A9^+^* and *SPP1^+^MMP9^+^* macrophage subpopulations, consistent with progressive glutamine-associated metabolic remodeling during macrophage transition. **(E)** Spatial distribution of *GLUL* expression: UMAP density plot showing the concentrated expression of *GLUL* (Glutamine Synthetase) within the myeloids clusters, particularly highlighting the metabolic hub of the transitional state. **(F)** *In vivo* validation of glutamine levels: Bar chart quantifying glutamine concentration in lung tissue from the murine bleomycin (BLM) model. Levels significantly increased by day 14 (D14) post-injury compared to vehicle controls, followed by a decline by day 28 (D28). **(G)** Representative multiplex immunofluorescence image of mouse lung tissue at day 0 and 14 post-bleomycin treatment, showing S100a9 (green), the macrophage marker Cd68 (red), GLS (magenta), and DAPI (blue). Fluorescent signal was quantified and compared between groups. Scale bar: 100 μm. Data are presented as mean ± SEM. N = 3 mice per group, *p < 0.05 **p < 0.01 vs Vehicle.

Recent studies have revealed the important role of metabolic reprogramming in macrophage fate shifting (40–42). To investigate the metabolic properties of the macrophage subpopulations in IPF and control lungs, we performed metabolite-mediated intercellular communication analysis and metabolic pathway profiling. Using MEBOCOST to map metabolite-sensor networks, we identified that S100A8^+^S100A9^+^ serve as highly active hubs for both sending and receiving metabolic signals in the lung microenvironment (**Figures 7A, B**). A global analysis of these networks identified L-Glutamine as a central metabolite involved in extensive crosstalk between macrophage subsets and structural cells (**Figure 7C**). Evaluation of aggregated enzyme expression across metabolic pathways confirmed that the glutamine metabolic program is enriched in *S100A8^+^S100A9^+^SPP1^+^MMP9^+^* macrophages and is also detectable in *FABP4^+^MME^+^* precursor macrophages, suggesting progressive glutamine-associated metabolic remodeling during macrophage transition (**Figure 7D**). This was further supported by the high expression density of GLUL (Glutamine Synthetase) within these specific myeloids clusters on the UMAP projection (**Figure 7E**). The increase of Glutamine metabolism was further confirmed in mouse IPF lung (**Figures 7F, G**). To determine whether the alterations observed in lung macrophages are detectable systemically, we analyzed peripheral blood mononuclear cell (PBMC) datasets. Similar to the lung tissue, we observed a high number of metabolite-sensor communications in the peripheral blood, particularly involving classical and intermediate monocytes (**Supplementary Figure 11A, B**). A specific upregulation of the glutamine pathway interaction strength was observed in the circulation (**Supplementary Figure 11C**). Paralleling our findings in lung tissue, the glutamine metabolic process was largely driven by the monocyte population (**Supplementary Figure 11D**), which showed significantly increased expression of key glutamine metabolism enzymes, including GLUL (**Supplementary Figure 11E**).

To confirm if this transcriptomic shift corresponds to a functional metabolic change *in vivo*, we assessed glutamine levels in the bleomycin-induced pulmonary fibrosis mouse model. This analysis demonstrated a significant increase in glutamine concentration in fibrotic lung tissue on day 14 compared to vehicle controls (**Figure 7F**). Consistent with the peak expression of transitional markers, these glutamine levels showed a relative decline by day 28. Together, these data demonstrate that *S100A8^+^S100A9^+^* macrophages are characterized by a distinct metabolic reprogramming toward glutamine hypermetabolism during the active phase of fibrotic progression. The increase in total lung glutamine supports a tissue-level metabolic association, although does not by itself establish that this change is specifically driven by *S100A8^+^S100A9^+^* macrophages.

## Discussion

In this study, our integrated single-cell analysis identifies a distinct *S100A8^+^S100A9^+^* transitional macrophage subpopulation in our dataset that is strongly associated with IPF progression. These cells form a bridge between an early tissue-repair state (*FABP4^+^MME^+^*) and a terminally differentiated, matrix-remodeling subset (*SPP1^+^MMP9^+^*), thus delineating a clear trajectory of pro-fibrotic macrophage differentiation. We further provide evidence suggesting that these transitional cells may be associated with fibrosis through two key mechanisms: inferred amplification of AREG-EGFR signaling to structural cells and transcriptional features consistent with glutamine metabolic reprogramming.

Our findings refine current paradigms of macrophage plasticity in IPF. While previous studies identified discrete profibrotic subsets, our analysis provides additional evidence for their dynamic ontogeny and supports the interpretation that the *S100A8^+^S100A9^+^* state represents a transitional stage linking early reparative and terminal profibrotic macrophage programs. This expands the view of *S100A8/A9* as mere inflammatory damage-associated molecular patterns (DAMPs); here, their sustained expression is associated with injury signals with metabolic rewiring and fibrotic effector functions. However, *S100A8* and *S100A9* are not unique to IPF and are elevated in a variety of acute and chronic inflammatory lung diseases, suggesting that they may reflect a more general signal of lung injury rather than an IPF-specific signature. This limitation should be considered when interpreting their biomarker potential, and future applications may benefit from combining these markers with existing clinical indices or more IPF-specific markers. The observed association with glutamine-related metabolic features illuminates how immune cells can potentially contribute to fibrosis, a mechanism previously described in cancer (43–45) but now also implicated in IPF macrophages. Importantly, the current mouse validation supports spatial and metabolic associations but does not by itself establish direct causality for this macrophage state or the implicated signaling and metabolic pathways in fibrosis. However, because the integrated lung analysis combined scRNA-seq and snRNA-seq datasets using a unified preprocessing framework, platform-related effects cannot be completely excluded, particularly for macrophage subsets that were unevenly represented across datasets. Therefore, the estimated abundance and inferred functional properties of these subsets should be interpreted with caution and will require validation in additional independent cohorts.

The diagnostic and therapeutic implications are significant. Current IPF diagnosis relies on late-stage radiological features, leading to misclassification in over 30% of cases (7, 9–11). The signature of the transitional macrophage subset, including elevated plasma *S100A8/A9, FCN1, and TIMP1*, offers a potential biomarker for early diagnosis before irreversible structural damage occurs. Future longitudinal biomarker studies in larger patient cohorts will be needed to assess its clinical utility potential. From a therapeutic standpoint, this macrophage subset represents a multifaceted target. Its dual role as a putative metabolic hub (with inferred involvement in glutamine metabolism) and a signaling node (predicted AREG secretion and EGFR-pathway engagement) offers multiple intervention points upstream of established fibrosis. Targeting *S100A8/A9, GLUL*-mediated glutamine metabolism, or the EGF pathway could disrupt pathogenic circuits and halt disease progression. Future work including cell-specific depletion and lineage-tracing will be required to investigate the causal role of this macrophage subset, and spatial profiling would clarify anatomical relationships within the fibrotic niche.

In summary, this study identifies the *S100A8^+^S100A9^+^* macrophage as a distinct and contextually important hub in IPF that integrates inflammatory, metabolic, and fibrogenic signals. We find that its pro-fibrotic phenotype is associated in part with inferred activation of the AREG-EGFR signaling pathway. Together, these findings nominate this macrophage subset and the associated pathways as promising candidate therapeutic targets, opening novel avenues for interceptive medicine in IPF.

## Data Availability

The datasets presented in this study can be found in online repositories. The names of the repository/repositories and accession number(s) can be found in the article/**Supplementary Material**.

## Supplementary material

Supplementary Figure 1 Quality control of single-cell RNA-seq datasets. Violin plots show the distributions of detected genes (nFeature_RNA), total UMI counts (nCount_RNA), and mitochondrial gene percentage (percent.mt) for each sample in GSE122960 **(A)**, GSE159354 **(B)**, GSE136831 **(C)**, and GSE159585 **(D)**. Horizontal plots summarize dataset-specific percent.mt distributions, with dashed lines indicating 5% and 10% mitochondrial thresholds.

Supplementary Figure 2 Quality assessment and robustness evaluation of GeneNMF-derived macrophage metaprograms. **(A)** Violin plots with embedded boxplots show the mitochondrial gene fraction for macrophages from GSE122960, GSE136831, GSE159354, and GSE159585. Macrophages from GSE159585 showed generally low mitochondrial gene percentages, supporting the quality of the retained population. **(B)** Clustered heatmap displaying the distinct macrophage metaprograms identified by GeneNMF analysis. Representative marker genes, including FABP4^+^MME^+^, AREG^+^PID1^+^, SPP1^+^MMP9^+^, and S100A8^+^S100A9^+^, characterize each consensus program. Color intensity indicates the degree of similarity among metaprograms. **(C)** Scatter plot showing the robustness of macrophage metaprograms identified by GeneNMF across GSE122960, GSE136831 and GSE159354. The x-axis indicates mean similarity, and the y-axis indicates silhouette score. Dot size represents the number of programs, and color intensity indicates sample coverage. **(D)** Scatter plot showing the robustness of macrophage metaprograms identified by GeneNMF across all four datasets: GSE122960, GSE136831, GSE159354, and GSE159585.

Supplementary Figure 3 Selection of the optimal consensus metaprogram number in GeneNMF. Robustness metrics were compared across different candidate values for the number of consensus metaprograms (nMP = 4–10) to evaluate the stability of the identified macrophage gene programs. The line graph displays the dynamic trends of three evaluation metrics: mean sample coverage (red line), mean silhouette score (green line), and mean similarity (blue line). The vertical dashed line indicates the final selected value (nMP = 8), representing the optimal trade-off between clustering quality and biological representation. Silhouette score and mean similarity were computed across individual NMF gene programs assigned to each metaprogram based on their gene-weight profiles, rather than across individual cells or graph-based clusters.

Supplementary Figure 4 UMAP visualization and compositional analysis of IPF and control samples. **(A)** UMAP projection showing the transcriptomic profiles of all 430,206 cells, colored by their individual biological sample ID. The slight sample skewing in specific UMAP regions likely reflects biological differences in cell-type composition between control and IPF lungs and should be interpreted together with **Figures 1B, E** rather than as incomplete integration. **(B)** Stacked bar chart illustrating the heterogeneity in cellular composition across the four source GEO datasets prior to integration.

Supplementary Figure 5 UMAP delineation of myeloid heterogeneity and macrophage substructure. **(A)** UMAP projection of all myeloid cells, colored by their major subpopulation identity (dendritic cells, macrophages, monocytes). **(B)** UMAP visualization shows the 14 distinct subgroups resolved from the initial high-resolution clustering of the macrophage population. **(C**) Violin plots displaying the module scores of the eight NMF metaprograms (MP1–MP8) across the 14 macrophage subgroups, demonstrating the differential gene program activity used for annotation. **(D)** UMAP projection illustrates the final classification and distribution of the seven functionally annotated macrophage subpopulations.

Supplementary Figure 6 Changes of key mechanosensing genes in IPF. Two-dimensional plots showing the relative expression scores for key mechanosensing genes (*PIEZO1/2, NINJ1, GPR68* and *TMEM63A/B/C*) in control (green) and IPF (blue) samples along the pseudo time axis.

Supplementary Figure 7 Single-cell landscape of peripheral blood mononuclear cells (PBMCs) in IPF and controls. **(A)** UMAP projection showing the robust integration of transcriptional profiles across all individual biological specimens. **(B)** Stacked bar charts showing the proportional abundance of cell types, stratified by clinical status (Left: Control vs. IPF) and by dataset origin (Right). **(C)** Bar chart detailing the inter-sample compositional variance in cell populations across each individual donor.

Supplementary Figure 8 Exploratory peripheral blood analysis supports enrichment of monocyte-associated markers in IPF. **(A)** Violin plots display the expression of *S100A8, S100A9, FCN1*, and *TIMP1* across peripheral blood cell populations in Ctrl and IPF samples. Cell populations include B cells, classical monocytes, intermediate monocytes, non-classical monocytes, plasma cells, T cells, and an uncertainty group. This analysis is presented for descriptive comparison across cell populations. **(B)** Violin plots with overlaid boxplots show the mean per-sample expression of *TIMP1* and *FCN1* in peripheral blood monocytes from Ctrl and IPF subjects. Each dot represents one subject/sample, and *p* values indicate sample-level comparisons between groups.

Supplementary Figure 9 Expression of the 4-gene signature across peripheral blood cell populations. **(A)** Violin plots with boxplots show the mean per-sample expression of each gene in the 4-gene signature (*S100A8, S100A9, FCN1*, and *TIMP1*) across peripheral blood cell populations in Ctrl and IPF subjects. Each dot represents one sample, and *p* values for comparisons between groups are indicated above each cell population.

Supplementary Figure 10 Intercellular communication network of IPF lung. **(A)** Global pathway-level analysis in different cell types and macrophage subpopulations in IPF lungs were performed. **(B)** The epidermal growth factor (EGF) signaling pathway was significantly enriched in *S100A8^+^S100A9^+^* macrophages compared to *FABP4^+^MME^+^* and *SPP1^+^MMP9^+^* macrophages.

Supplementary Figure 11 Metabolic communication and reprogramming in peripheral blood mononuclear cells of IPF patients. **(A)** Quantitative analysis of peripheral metabolic communication: Bar chart displaying the number of metabolite-sensor interactions across peripheral blood cell types. **(B)** Global peripheral metabolite signaling heatmap: Dot plot illustrating the interaction scores between sender and receiver cell populations in the blood. The dot size corresponds to the number of communication events, while color indicates the overall communication score. **(C)** Peripheral metabolite-sensor interaction network: Chord diagram mapping the connections between peripheral cell types, specific metabolites, and their corresponding sensors. L-Glutamine (red box) is highlighted as a primary metabolite involved in systemic crosstalk. **(D)** Cell-specific metabolic enzyme expression in PBMCs: Violin plots showing the mean aggregated enzyme expression for various metabolic pathways across peripheral cell types. L-Glutamine metabolism (red box) is specifically enriched in Monocyte classical and Monocyte intermediate subsets. **(E)** Spatial distribution of *GLUL* expression in circulating cells: UMAP density plot showing that *GLUL* (Glutamine Synthetase) expression is primarily concentrated within the monocyte clusters of the peripheral blood.
