# Supplementary figures and images for: S100A8+S100A9+ transitional macrophages are associated with pulmonary fibrosis progression by integrating immunometabolism and fibrogenic crosstalk
Source: Front Immunol. 2026 Jul 2;17:1832940. doi: 10.3389/fimmu.2026.1832940 (PMC13372650; doi:10.3389/fimmu.2026.1832940)

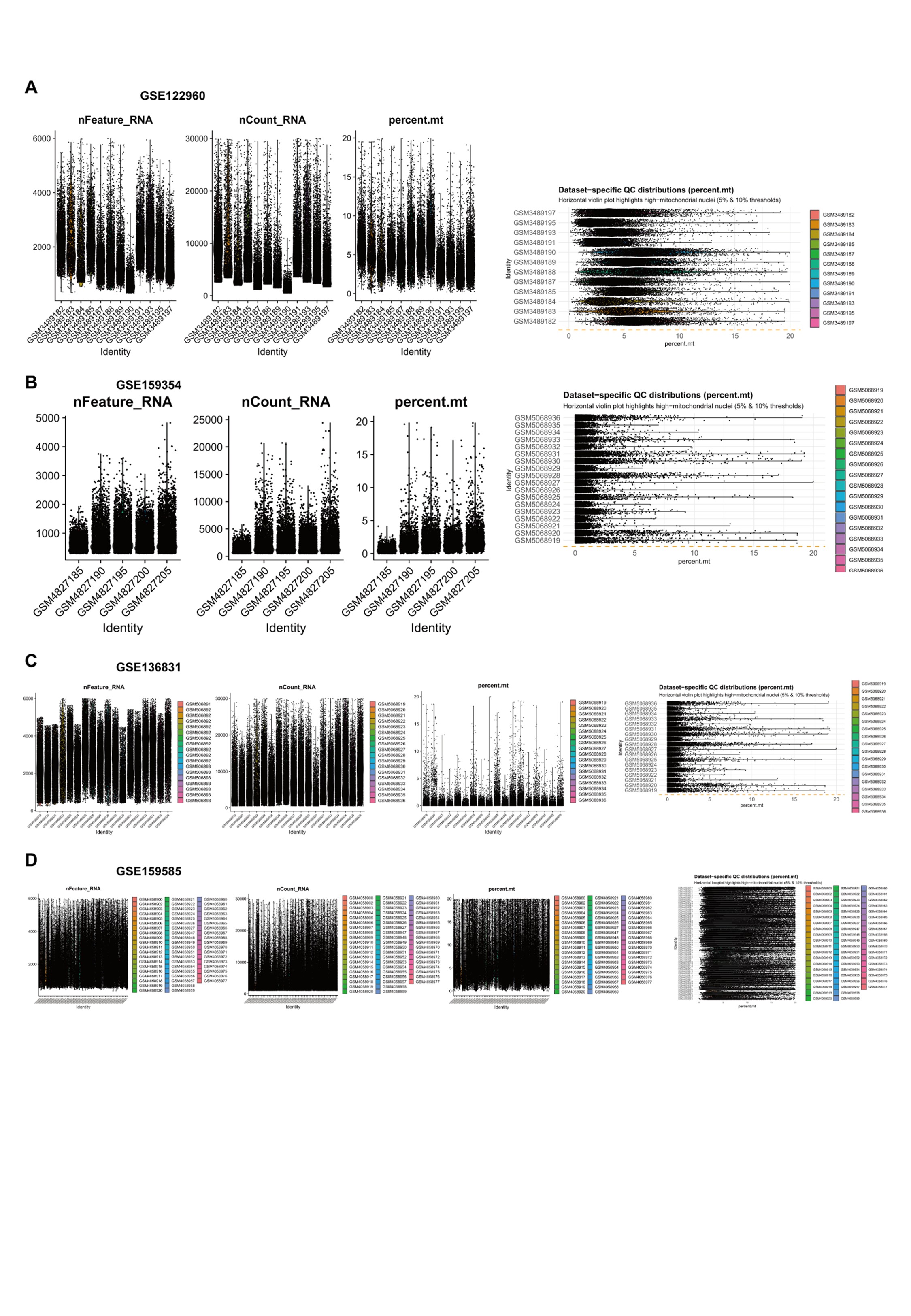

Supplement: Supplementary Figure 1 — Quality control of single-cell RNA-seq datasets. Violin plots show the distributions of detected genes (nFeature_RNA), total UMI counts (nCount_RNA), and mitochondrial gene percentage (percent.mt) for each sample in GSE122960 (A), GSE159354 (B), GSE136831 (C), and GSE159585 (D). Horizontal plots summarize dataset-specific percent.mt distributions, with dashed lines indicating 5% and 10% mitochondrial thresholds. [file Image1.jpeg]

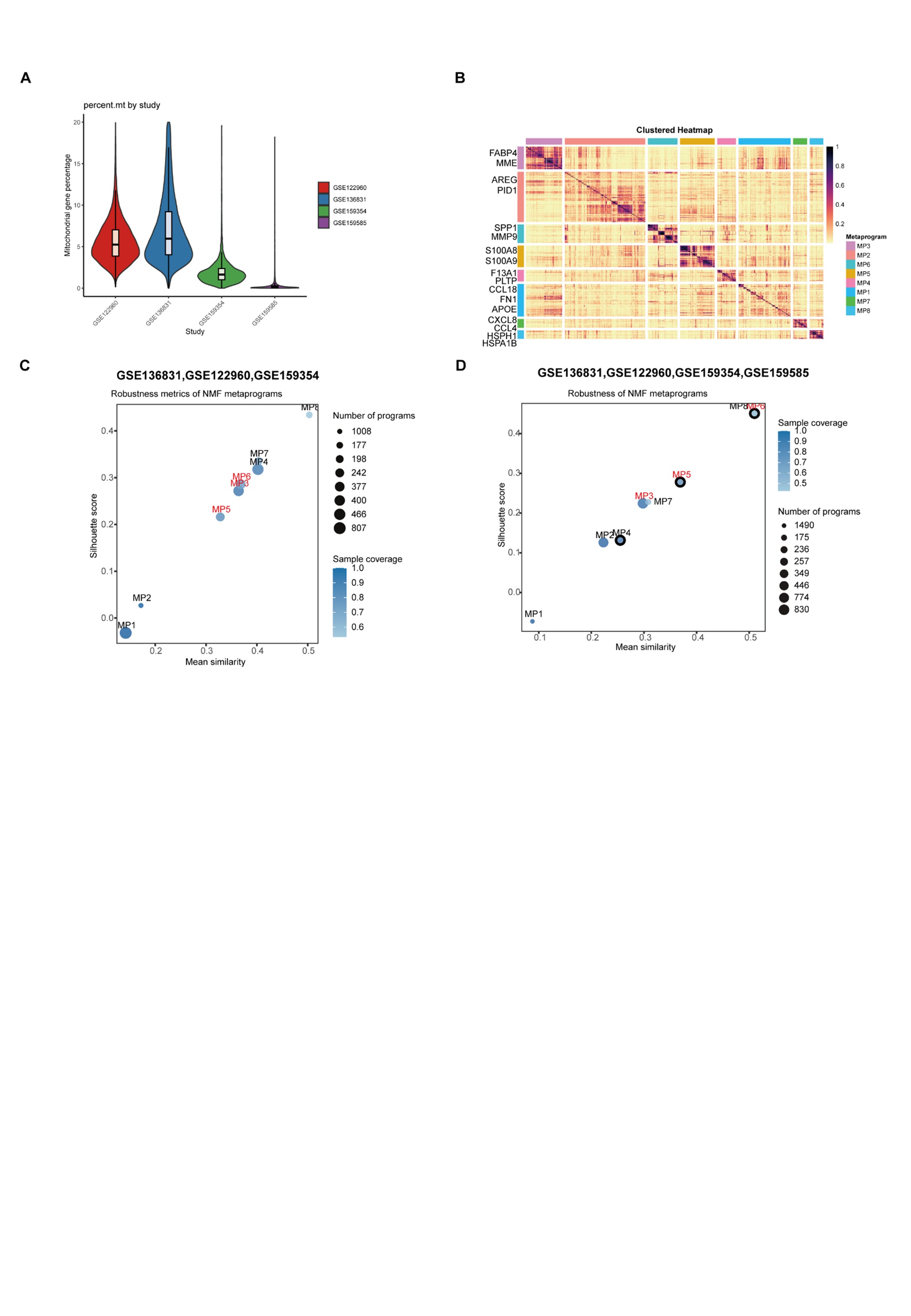

Supplement: Supplementary Figure 2 — Quality assessment and robustness evaluation of GeneNMF-derived macrophage metaprograms. (A) Violin plots with embedded boxplots show the mitochondrial gene fraction for macrophages from GSE122960, GSE136831, GSE159354, and GSE159585. Macrophages from GSE159585 showed generally low mitochondrial gene percentages, supporting the quality of the retained population. (B) Clustered heatmap displaying the distinct macrophage metaprograms identified by GeneNMF analysis. Representative marker genes, including FABP4+MME+, AREG+PID1+, SPP1+MMP9+, and S100A8+S100A9+, characterize each consensus program. Color intensity indicates the degree of similarity among metaprograms. (C) Scatter plot showing the robustness of macrophage metaprograms identified by GeneNMF across GSE122960, GSE136831 and GSE159354. The x-axis indicates mean similarity, and the y-axis indicates silhouette score. Dot size represents the number of programs, and color intensity indicates sample coverage. (D) Scatter plot showing the robustness of macrophage metaprograms identified by GeneNMF across all four datasets: GSE122960, GSE136831, GSE159354, and GSE159585. [file Image2.jpeg]

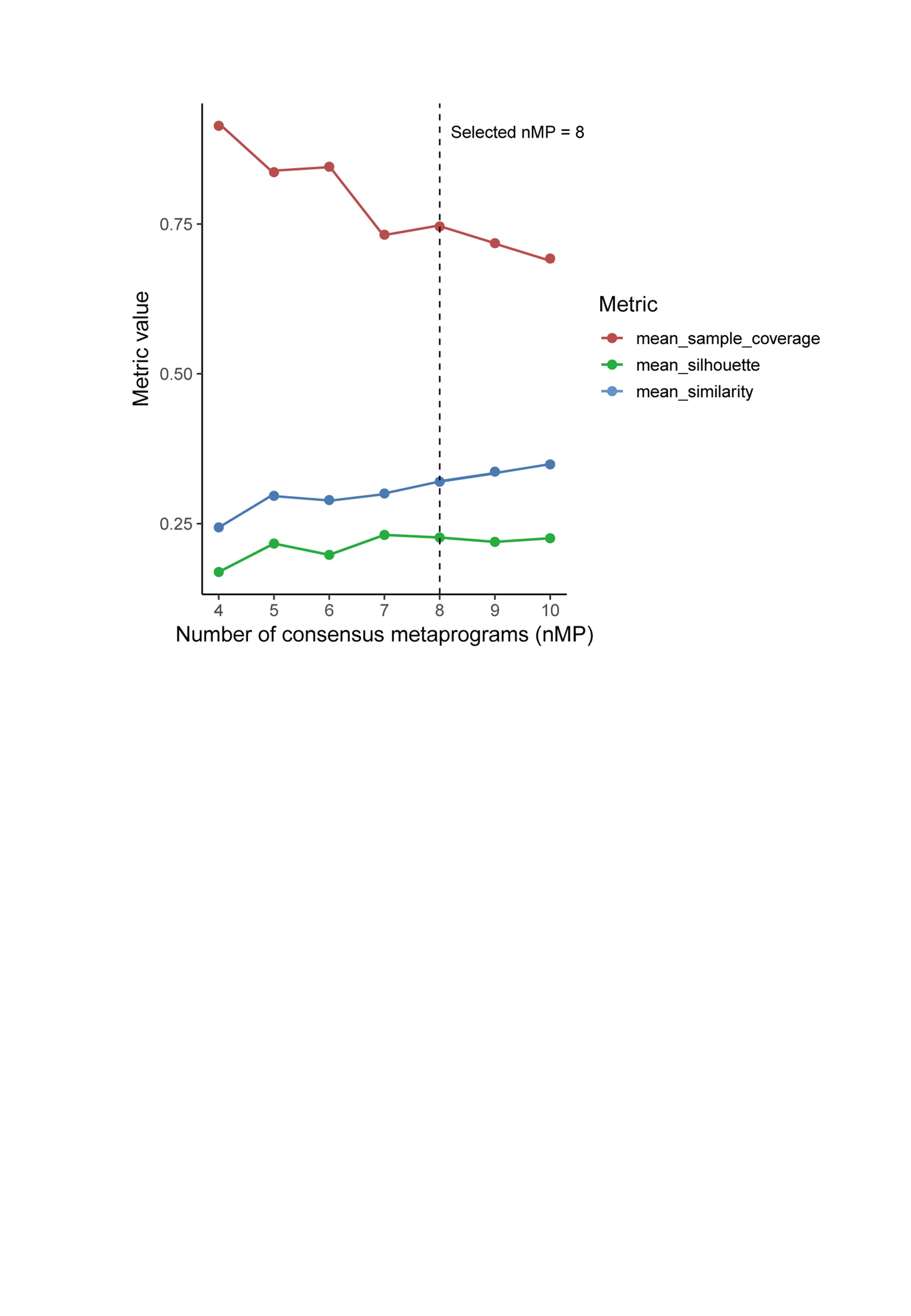

Supplement: Supplementary Figure 3 — Selection of the optimal consensus metaprogram number in GeneNMF. Robustness metrics were compared across different candidate values for the number of consensus metaprograms (nMP = 4–10) to evaluate the stability of the identified macrophage gene programs. The line graph displays the dynamic trends of three evaluation metrics: mean sample coverage (red line), mean silhouette score (green line), and mean similarity (blue line). The vertical dashed line indicates the final selected value (nMP = 8), representing the optimal trade-off between clustering quality and biological representation. Silhouette score and mean similarity were computed across individual NMF gene programs assigned to each metaprogram based on their gene-weight profiles, rather than across individual cells or graph-based clusters. [file Image3.jpeg]

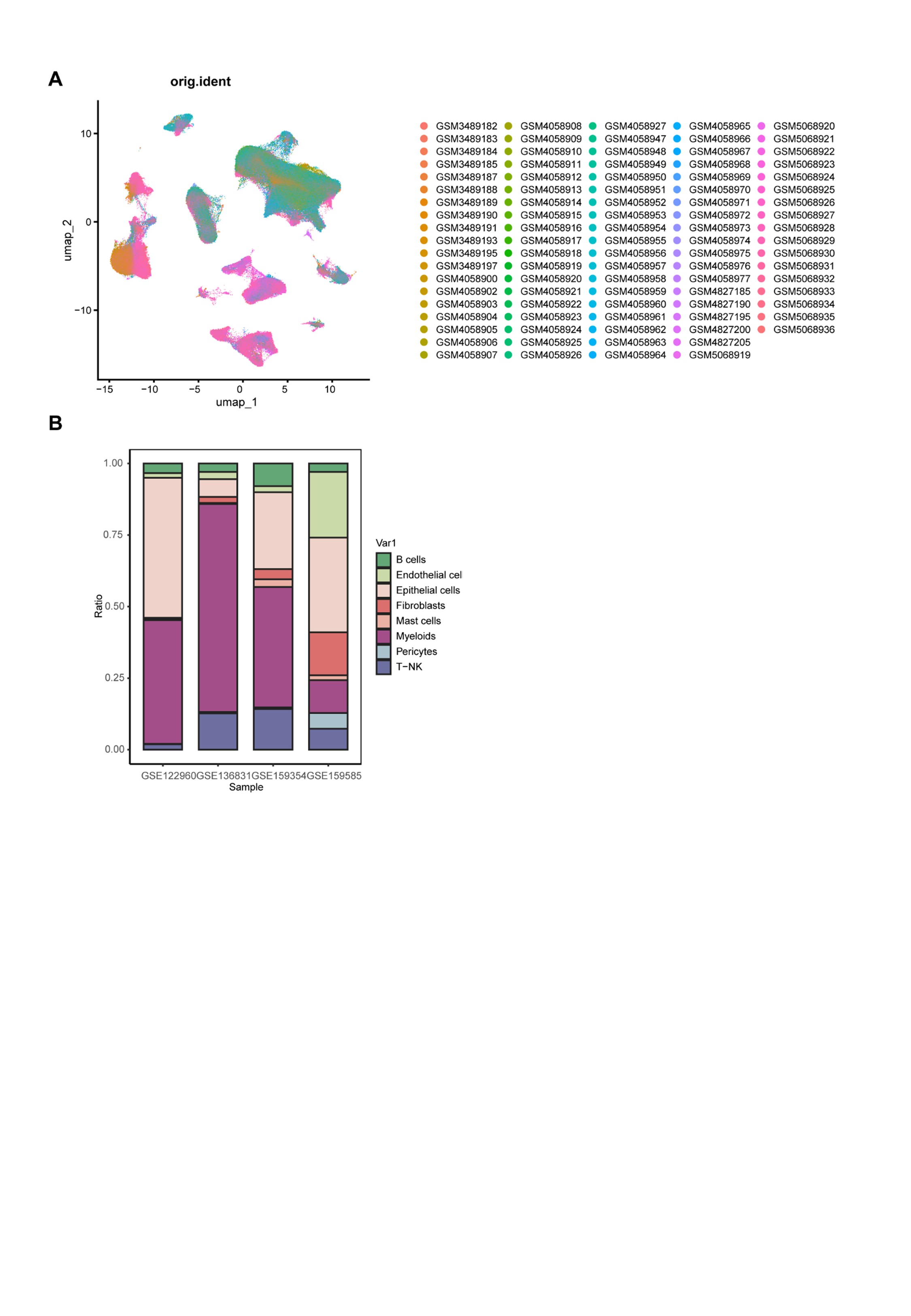

Supplement: Supplementary Figure 4 — UMAP visualization and compositional analysis of IPF and control samples. (A) UMAP projection showing the transcriptomic profiles of all 430,206 cells, colored by their individual biological sample ID. The slight sample skewing in specific UMAP regions likely reflects biological differences in cell-type composition between control and IPF lungs and should be interpreted together with Figures 1B, E rather than as incomplete integration. (B) Stacked bar chart illustrating the heterogeneity in cellular composition across the four source GEO datasets prior to integration. [file Image4.jpeg]

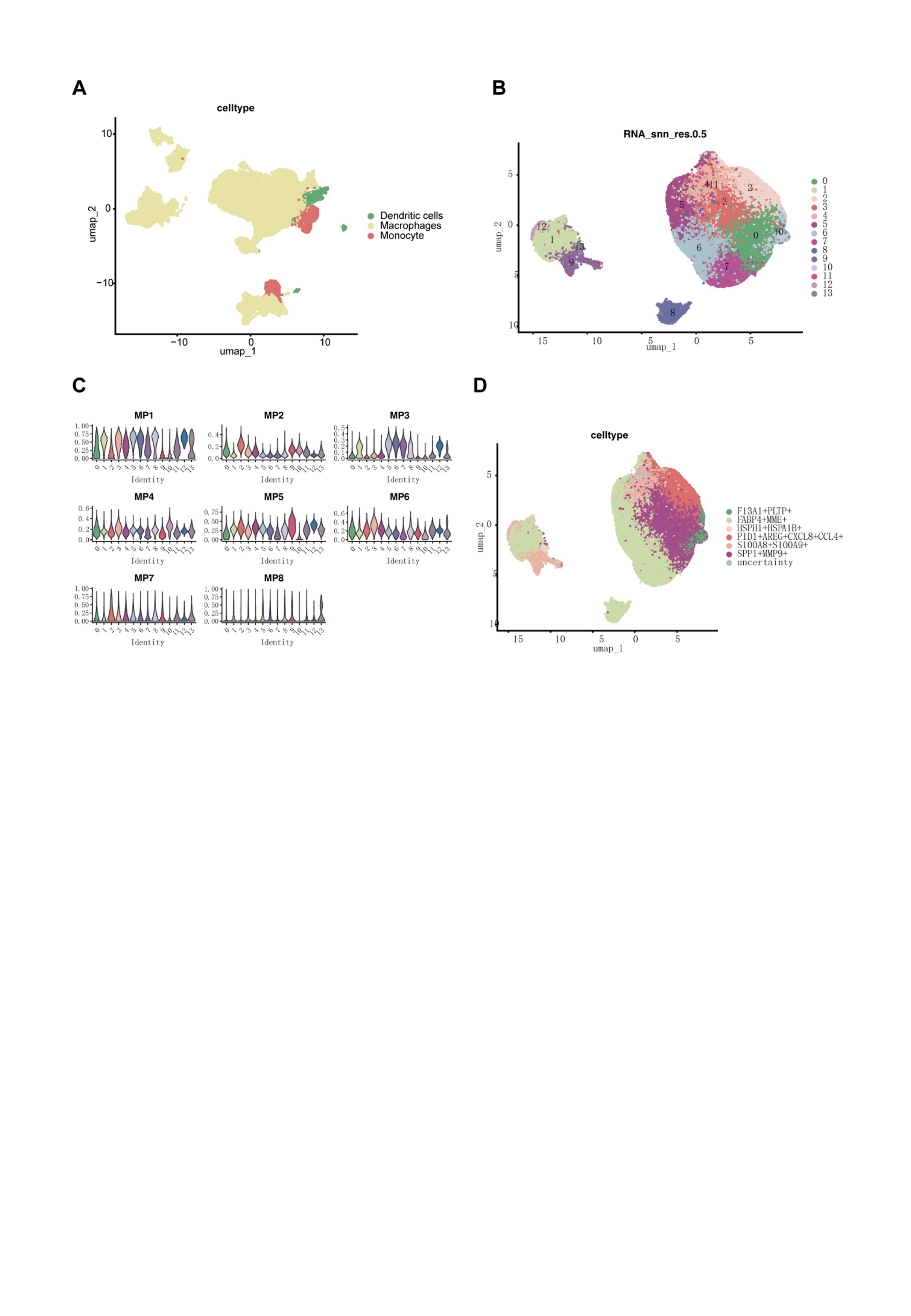

Supplement: Supplementary Figure 5 — UMAP delineation of myeloid heterogeneity and macrophage substructure. (A) UMAP projection of all myeloid cells, colored by their major subpopulation identity (dendritic cells, macrophages, monocytes). (B) UMAP visualization shows the 14 distinct subgroups resolved from the initial high-resolution clustering of the macrophage population. (C) Violin plots displaying the module scores of the eight NMF metaprograms (MP1–MP8) across the 14 macrophage subgroups, demonstrating the differential gene program activity used for annotation. (D) UMAP projection illustrates the final classification and distribution of the seven functionally annotated macrophage subpopulations. [file Image5.jpeg]

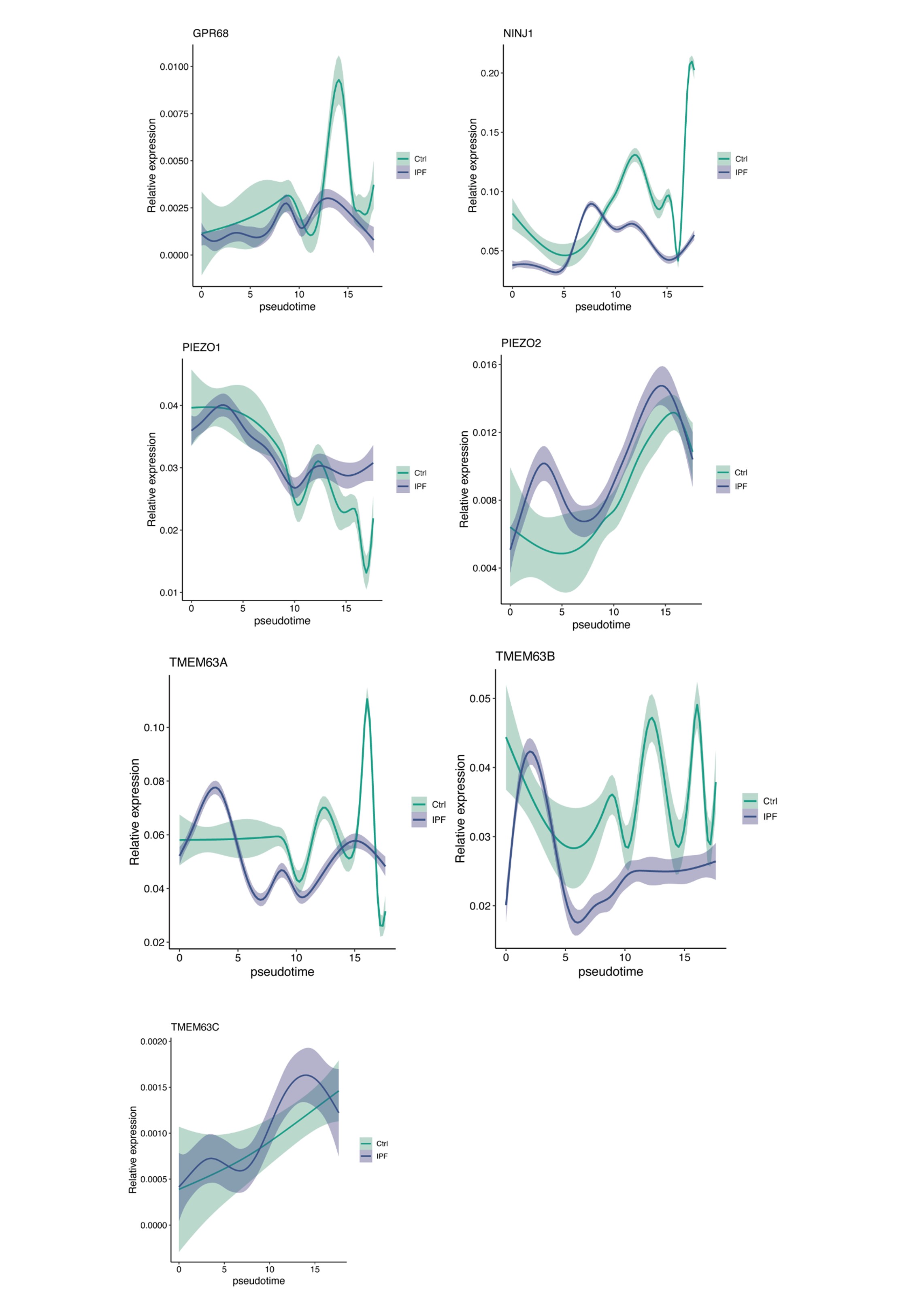

Supplement: Supplementary Figure 6 — Changes of key mechanosensing genes in IPF. Two-dimensional plots showing the relative expression scores for key mechanosensing genes (PIEZO1/2, NINJ1, GPR68 and TMEM63A/B/C) in control (green) and IPF (blue) samples along the pseudo time axis. [file Image6.jpeg]

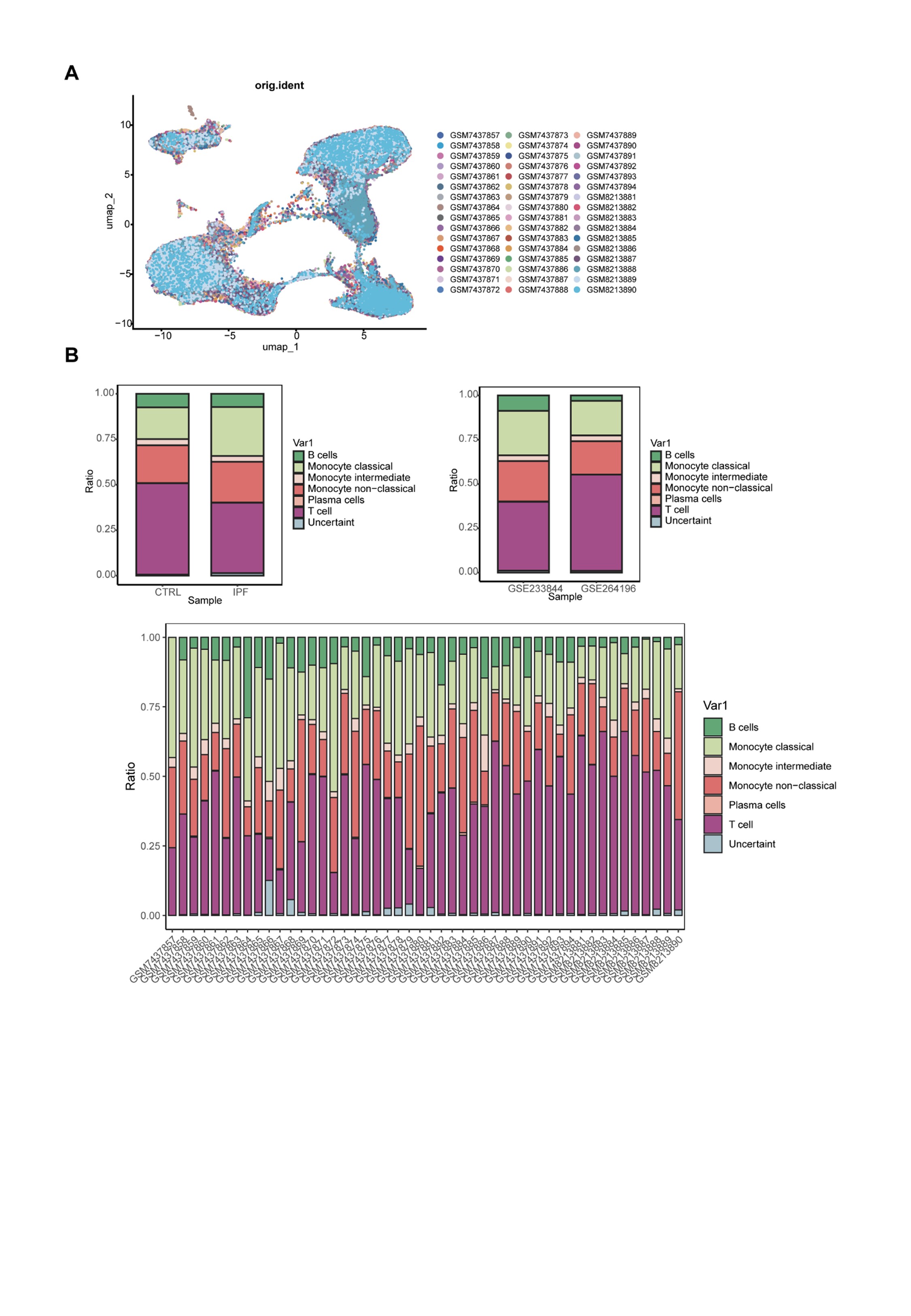

Supplement: Supplementary Figure 7 — Single-cell landscape of peripheral blood mononuclear cells (PBMCs) in IPF and controls. (A) UMAP projection showing the robust integration of transcriptional profiles across all individual biological specimens. (B) Stacked bar charts showing the proportional abundance of cell types, stratified by clinical status (Left: Control vs. IPF) and by dataset origin (Right). (C) Bar chart detailing the inter-sample compositional variance in cell populations across each individual donor. [file Image7.jpeg]

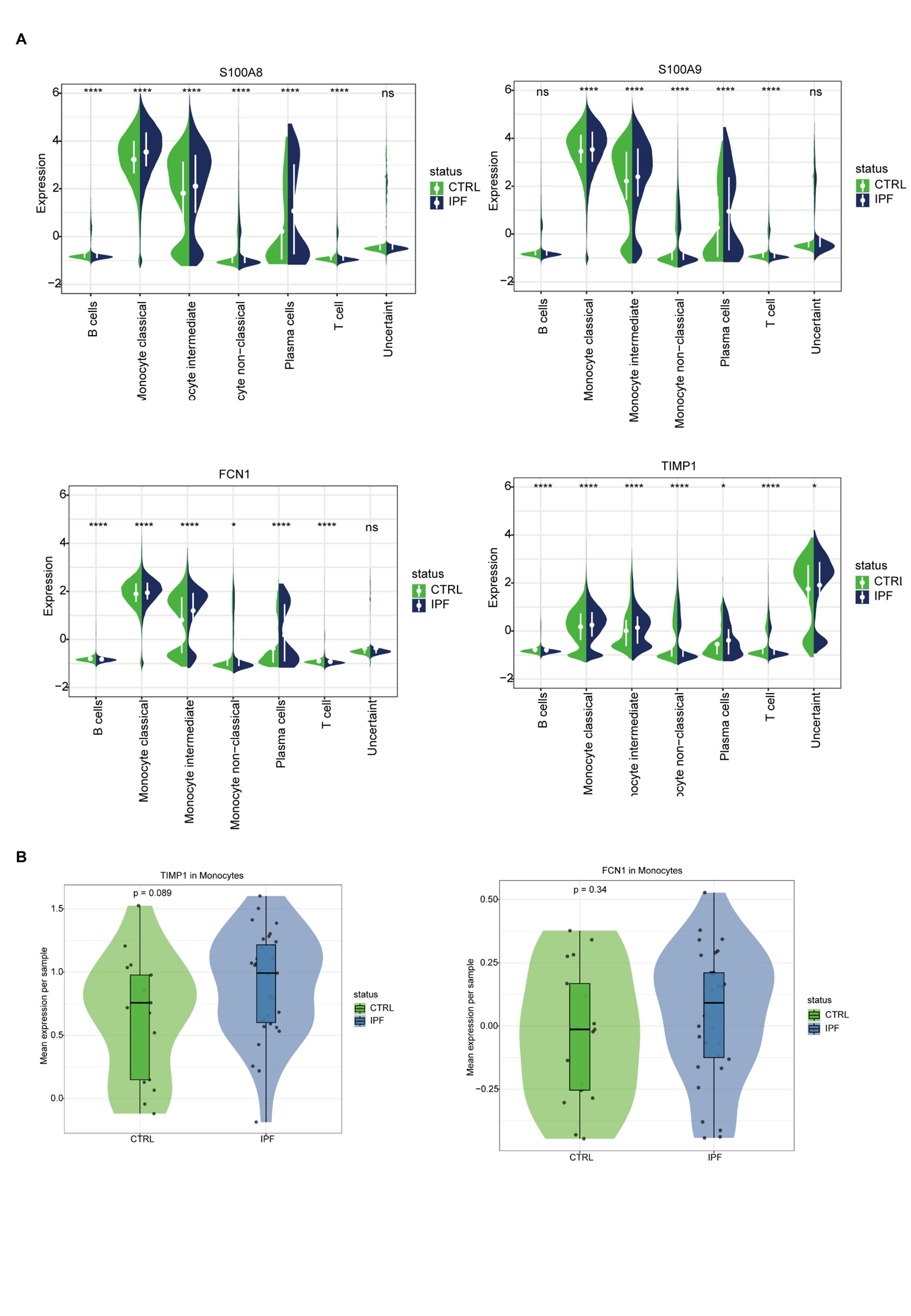

Supplement: Supplementary Figure 8 — Exploratory peripheral blood analysis supports enrichment of monocyte-associated markers in IPF. (A) Violin plots display the expression of S100A8, S100A9, FCN1, and TIMP1 across peripheral blood cell populations in Ctrl and IPF samples. Cell populations include B cells, classical monocytes, intermediate monocytes, non-classical monocytes, plasma cells, T cells, and an uncertainty group. This analysis is presented for descriptive comparison across cell populations. (B) Violin plots with overlaid boxplots show the mean per-sample expression of TIMP1 and FCN1 in peripheral blood monocytes from Ctrl and IPF subjects. Each dot represents one subject/sample, and p values indicate sample-level comparisons between groups. [file Image8.jpg]

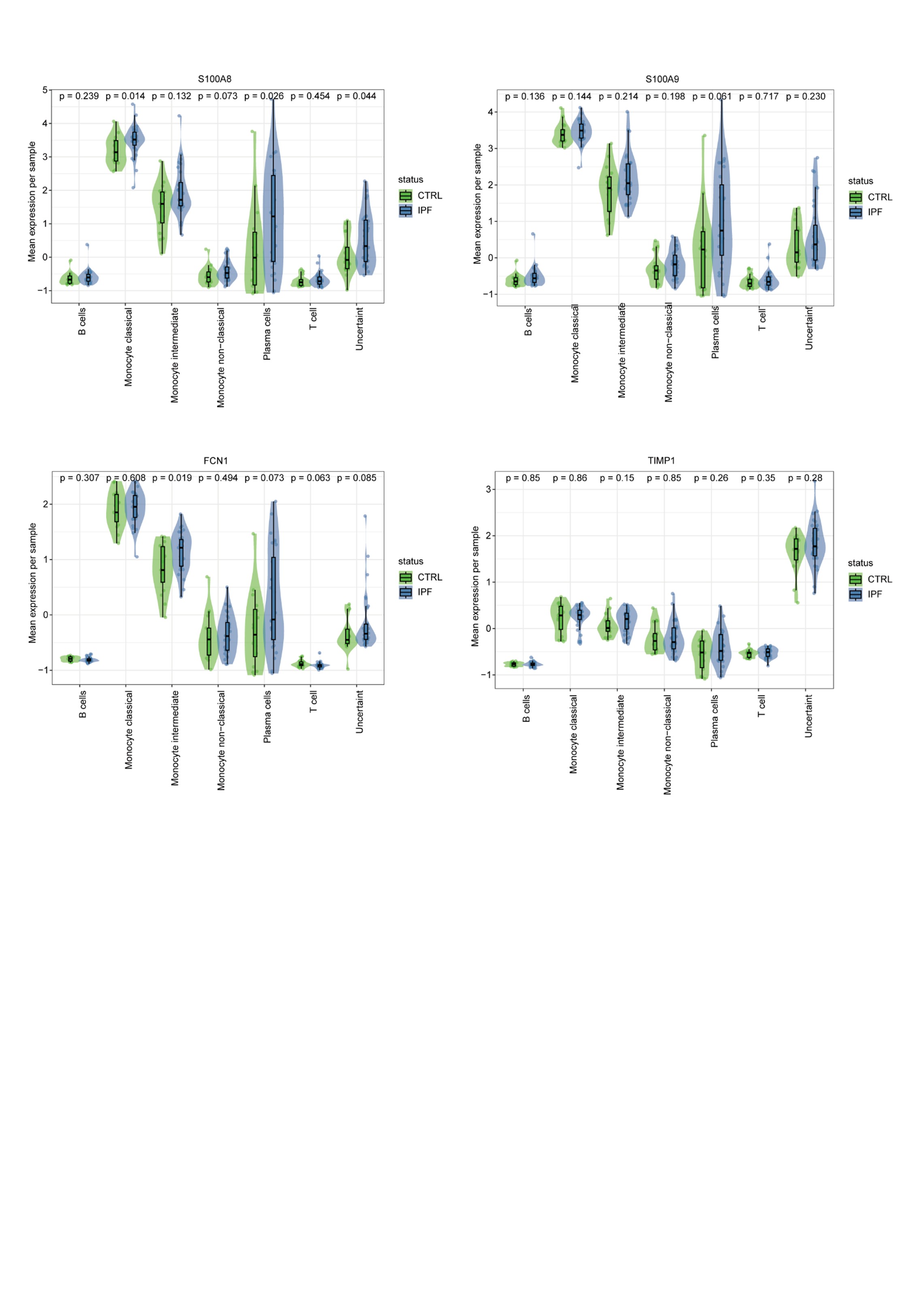

Supplement: Supplementary Figure 9 — Expression of the 4-gene signature across peripheral blood cell populations. (A) Violin plots with boxplots show the mean per-sample expression of each gene in the 4-gene signature (S100A8, S100A9, FCN1, and TIMP1) across peripheral blood cell populations in Ctrl and IPF subjects. Each dot represents one sample, and p values for comparisons between groups are indicated above each cell population. [file Image9.jpeg]

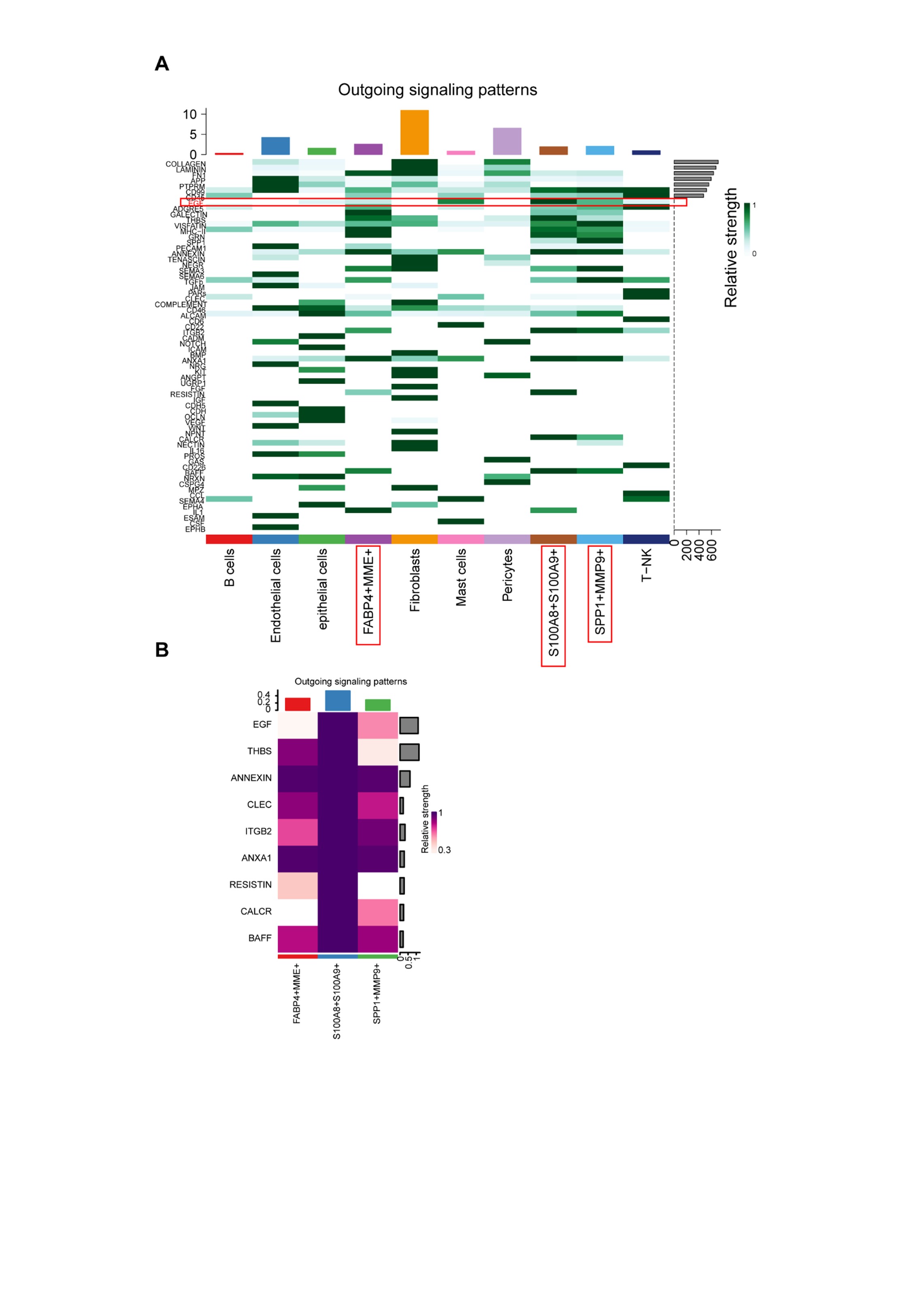

Supplement: Supplementary Figure 10 — Intercellular communication network of IPF lung. (A) Global pathway-level analysis in different cell types and macrophage subpopulations in IPF lungs were performed. (B) The epidermal growth factor (EGF) signaling pathway was significantly enriched in S100A8+S100A9+ macrophages compared to FABP4+MME+ and SPP1+MMP9+ macrophages. [file Image10.jpeg]

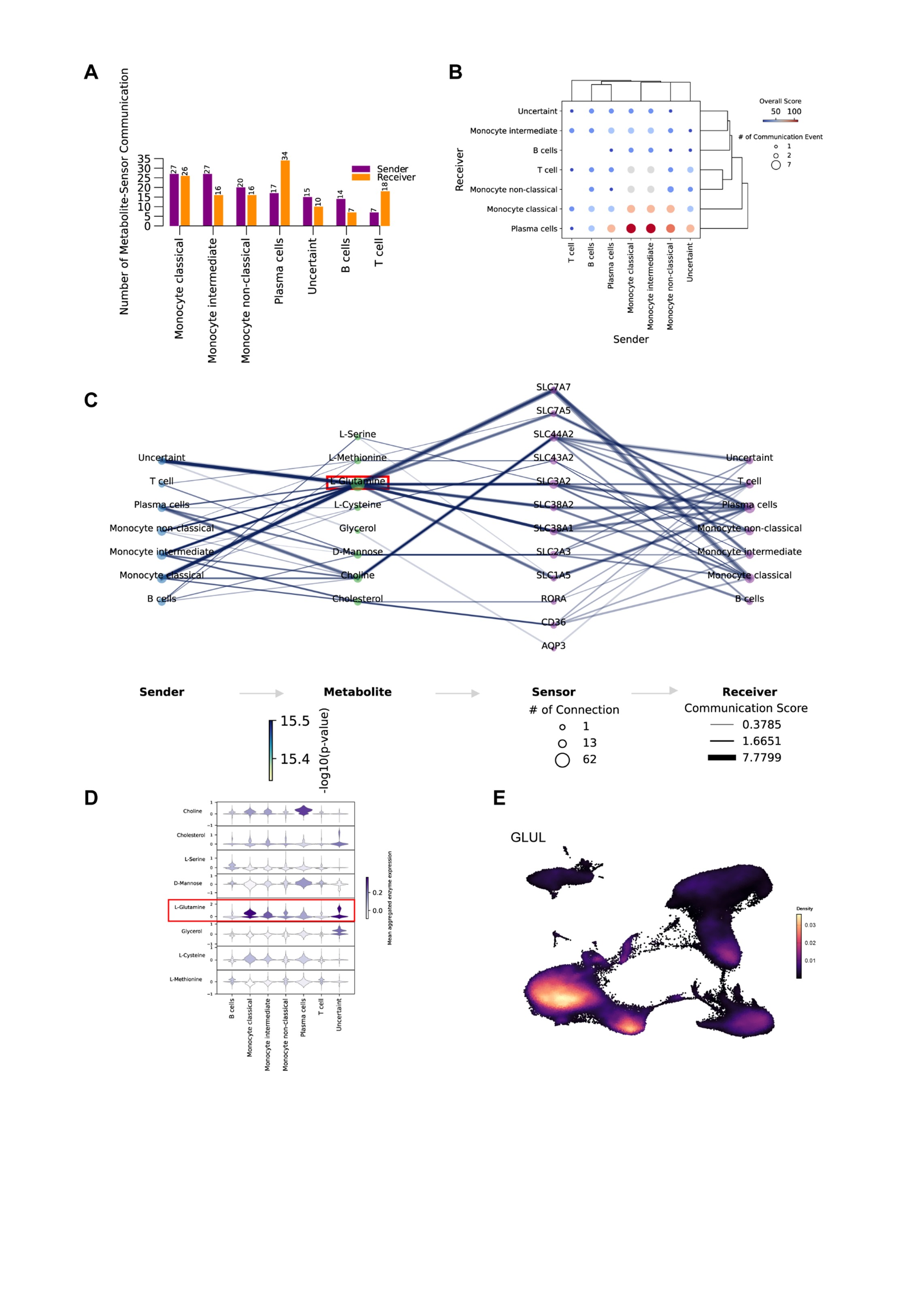

Supplement: Supplementary Figure 11 — Metabolic communication and reprogramming in peripheral blood mononuclear cells of IPF patients. (A) Quantitative analysis of peripheral metabolic communication: Bar chart displaying the number of metabolite-sensor interactions across peripheral blood cell types. (B) Global peripheral metabolite signaling heatmap: Dot plot illustrating the interaction scores between sender and receiver cell populations in the blood. The dot size corresponds to the number of communication events, while color indicates the overall communication score. (C) Peripheral metabolite-sensor interaction network: Chord diagram mapping the connections between peripheral cell types, specific metabolites, and their corresponding sensors. L-Glutamine (red box) is highlighted as a primary metabolite involved in systemic crosstalk. (D) Cell-specific metabolic enzyme expression in PBMCs: Violin plots showing the mean aggregated enzyme expression for various metabolic pathways across peripheral cell types. L-Glutamine metabolism (red box) is specifically enriched in Monocyte classical and Monocyte intermediate subsets. (E) Spatial distribution of GLUL expression in circulating cells: UMAP density plot showing that GLUL (Glutamine Synthetase) expression is primarily concentrated within the monocyte clusters of the peripheral blood. [file Image11.jpeg]
